# Two *KCNQ2* Encephalopathy Variants in the Calmodulin-Binding Helix A Exhibit Dominant-Negative Effects and Altered PIP_2_ Interaction

**DOI:** 10.3389/fphys.2020.571813

**Published:** 2020-09-11

**Authors:** Baouyen Tran, Zhi-Gang Ji, Mingxuan Xu, Tammy N. Tsuchida, Edward C. Cooper

**Affiliations:** ^1^Department of Neuroscience, Baylor College of Medicine, Houston, TX, United States; ^2^Department of Neurology, Baylor College of Medicine, Houston, TX, United States; ^3^Departments of Pediatrics and Neurology, Children’s National Medical Center, Washington, DC, United States; ^4^Department of Molecular and Human Genetics, Baylor College of Medicine, Houston, TX, United States

**Keywords:** SF0034, Kv7.2, epileptic encephalopathy, phosphatidylinositol 4,5-bisphosphate, calmodulin

## Abstract

Heterozygous missense variants in *KCNQ2*, which encodes the potassium channel subunit Kv7.2, are among the most common genetic causes of severe neonatal-onset epileptic encephalopathy. Because about 20% of known severe Kv7.2 missense changes lie within the intracellular C-terminal region, improving understanding of the underlying pathogenic mechanisms is important. We analyzed the basis for the severe phenotypes of Kv7.2 A337T and A337G, variants in the C-terminal’s calmodulin (CaM)-binding Helix A. When expressed heterologously in mammalian cells, alone or in combination with wild type Kv7.2 or with wild type Kv7.2 and Kv7.3, both variants strongly suppressed channel currents. A337T channels expressed alone exhibited significantly reduced protein half-life and surface trafficking and co-immunoprecipitated less CaM. For both variants, increasing cellular phosphatidylinositol 4,5-bisphosphate (PIP_2_) by overexpression of PI(4)P5-kinase restored current densities. For both variants, the fraction of current suppressed by activation of M1 muscarinic receptors with 10 μM oxotremorine methiodide, which depletes PIP_2_, was less than for controls. During voltage-sensitive phosphatase-induced transient PIP_2_ depletion and resynthesize, potassium current inhibition and recovery kinetics were both markedly slowed. These results suggest that these variants may reduce currents by a mechanism not previously described: slowing of PIP_2_ migration between the bulk membrane and binding sites mediating channel electromechanical coupling. A novel Kv7.2/3-selective opener, SF0034, rescued current amplitudes. Our findings show that these two Helix A variants suppress channel current density strongly, consistent with their severe heterozygous phenotypes, implicate impairment of CaM and PIP_2_ regulation in *KCNQ2* encephalopathy pathogenesis, and highlight the potential usefulness of selective Kv7 openers for this distinctive pathogenic mechanism and patient subgroup.

## Introduction

Variants in *KCNQ2* can underlie a severe syndrome consisting of intractable neonatal onset seizures and profound global developmental delay called *KCNQ2* encephalopathy (Weckhuysen et al., 2012; Millichap et al., 2016). Indeed, recent cohort studies have shown that 10–30% of previously undiagnosed patients with early infantile epileptic encephalopathy (EIEE) have *de novo* heterozygous missense or single codon deletion variants in *KCNQ2* (Saitsu et al., 2012; Weckhuysen et al., 2012; Kato et al., 2013; Milh et al., 2013; Olson et al., 2017). *KCNQ2* was first discovered at the locus for a mild, self-limiting neonatal syndrome, benign familial neonatal epilepsy (BFNE) (Biervert et al., 1998; Charlier et al., 1998; Singh et al., 1998). *KCNQ2* variants causing BFNE are usually inherited as an autosomal dominant trait with high penetrance but sometimes appears *de novo*. It is important to understand *KCNQ2* genotype-phenotype relationships so that individuals with BFNE or with rare non-pathogenic variants can be rapidly distinguished from those with EIEE-causing variants. BFNE infants have a good prognosis, and ill infants with non-pathogenic *KCNQ2* variants need further diagnostic efforts. Individuals with pathogenic *KCNQ2* encephalopathy variants have ended their diagnostic odyssey, have a poor long-term prognosis, and are appropriate candidates for novel therapeutic trials.

**Figure 1:**
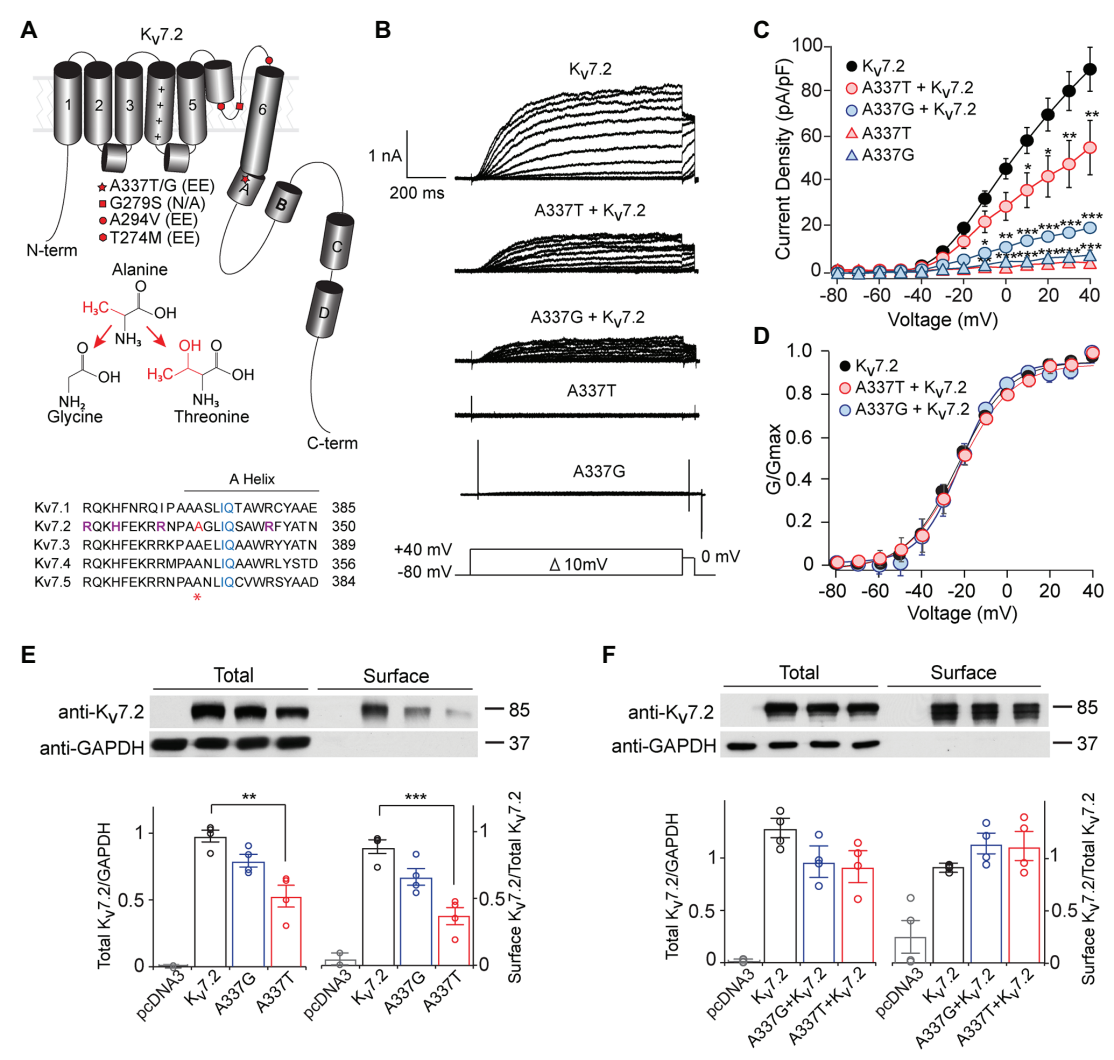
A337T and A337G suppressed Kv7.2 currents. **(A)** Cartoon showing Kv7.2 membrane topology, highlighting positions of functionally important α-helical segments and domains, and the variants analyzed in this study. The side chain changes resulting from the two clinical variants at A337 are shown. Alignment of human *KCNQ* sequences around *KCNQ2* A337 (red and asterisk) shows high conservation across the gene family. Basic residues previously implicated in PIP_2_ binding (Zhang et al., 2013; Kim et al., 2016a; Soldovieri et al., 2016; purple) and the canonical CaM binding IQ motif (blue), are highlighted. A337 (highlighted in red and asterisk) is near the proximal end of Helix A. **(B)** Representative currents from Chinese hamster ovary (CHO) cells transfected with WT Kv7.2, A337G, and A337T in response to depolarization steps from −80 mV to between −70 and +40 mV. **(C)** Current-voltage plots show significant current reductions in cells expressing either A337T or A337G (^**^p < 0.01, ^***^p < 0.001, Two-way ANOVA, Bonferroni test, *n* = 14–31, see Table 1). **(D)** Conductance-voltage plots show no significant differences of WT and mutant-containing channels (see Table 1). **(E)** Western blots of cell lysates (left lanes, ^**^p = 0.003, One-way ANOVA, Tukey test) and surface biotinylated proteins (right lanes, ^***^p < 0.001, One-way ANOVA, Tukey test) from CHO cells electroporated with empty pcDNA3 vector (negative control), Kv7.2, A337G or A337T (*n* = 4). In plots, quantification of total channel protein normalized to GAPDH (left) and surface channel protein normalized to total channel protein are shown. Values are normalized to WT. **(F)** Western blot of cell lysates (left lanes) and surface membrane biotinylated proteins (right lanes) from CHO cells electroporated with WT Kv7.2 alone or with 1:1 ratio of Kv7.2 and A337G or A337T cDNAs (*n* = 4). The cropped blot images are from full-length gels shown as supplementary information (Supplementary Figure S2).

*KCNQ2* and its close homolog, *KCNQ3*, encode potassium channel subunits Kv7.2 and Kv7.3, respectively, which form tetrameric neuronal voltage-gated potassium channels. Both homomers (formed by Kv7.2 alone) and heteromers (combining Kv7.2 and Kv7.3) underlie the neuronal M-current (I_M_), a voltage-dependent, slowly activating, non-inactivating current regulated by G_q_-coupled receptors including M1 muscarinic acetylcholine receptors (M1Rs; Wang et al., 1998; Delmas and Brown, 2005). These channels are broadly expressed in the central nervous system, where they are highly concentrated at axon initial segments (AISs) and nodes of Ranvier, and can influence action potential generation and conduction (Cooper et al., 2000; Devaux et al., 2004; Pan et al., 2006; Shah et al., 2008; Battefeld et al., 2014). Kv7.2 and Kv7.3 have a classical 6-transmembrane voltage-gated potassium channel topology but possess a distinctive, large intracellular CT domain (Figure 1A). Known roles of the CT include integrating modulatory interactions, including with calmodulin (CaM; Wen and Levitan, 2002; Yus-Nájera et al., 2002) and phosphatidylinositol 4,5-bisphosphate (PIP_2_; Zhang et al., 2003; Li et al., 2005; Brown et al., 2007; Gamper and Shapiro, 2007; Hernandez et al., 2008; Zaydman and Cui, 2014).

Considerable evidence indicates that CaM and PIP_2_ interactions are allosterically coupled (Kosenko et al., 2012; Kosenko and Hoshi, 2013; Zaydman and Cui, 2014; Alberdi et al., 2015; Chen et al., 2015). Although Kv7.1 atomic structures have recently been obtained in the presence of CaM, with and without bound PIP_2_ (Sun and MacKinnon, 2017, 2020), dynamic mechanisms of Ca^2+^-CaM and PIP_2_ regulation remain poorly understood. CaM binding at two α-helical segments of the Kv7.2 CT domain, Helix A and B, has been proposed to be required for ER exit and targeting to the axon (Etxeberria et al., 2008; Alaimo et al., 2009; Cavaretta et al., 2014; Liu and Devaux, 2014). PIP_2_ binding is required for the voltage-dependent opening of Kv7 channels and appears to contribute directly to coupling voltage sensor movement and pore gate opening (Zaydman et al., 2013; Zaydman and Cui, 2014; Sun and MacKinnon, 2017, 2020). However, sites functionally implicated in Kv7 channel PIP_2_ modulation extend beyond the binding pocket seen by cryoelectron microscopy and include conserved H, K, and R residues from the S2–3 linker, S4–5 linker, S6, the CT region upstream of the Helix A, and on linkers between the A, B, and C helices (Hernandez et al., 2008; Telezhkin et al., 2013; Zaydman and Cui, 2014; Hille et al., 2015).

Most BFNE missense variants cause mild loss-of-function when co-expressed with wild type (WT) subunits, whereas most EIEE variants cause severe dominant-negative suppression of activity. However, only a small proportion of known variants have been studied to date. Previous studies of human Kv7.2 variants from the CT domain have mostly focused on those causing BFNE (Richards et al., 2004; Alaimo et al., 2009; Orhan et al., 2014; Alberdi et al., 2015; Ambrosino et al., 2015). Recently, a heterozygous Kv7.2 EIEE variant in the proximal CT, R325G, was shown to suppress currents by directly impairing PIP_2_ binding (Soldovieri et al., 2016). Here, we analyze the functional consequences of A337T and A337G, two *KCNQ2* encephalopathy variants (Saitsu et al., 2012; Millichap et al., 2016), at a conserved residue in the CaM-binding Helix A (Figure 1A). The pathogenicity of these variants has remained less certain due to rarity: To date, only one individual has been described with each. We found that both variants cause loss of current amplitude sufficient to be considered as dominant-negative, supporting the clinical diagnoses of *KCNQ2* encephalopathy. We obtained evidence that the suppression of Kv7.2 + Kv7.3 channel function involves a novel pattern of disruption of PIP_2_ and (for A337T) CaM interaction. Ezogabine was previously used successfully to control seizures and improve EEG in the infant with A337T (Millichap et al., 2016). We show that SF0034, a close ezogabine analog with improved potency and KCNQ2/3 selectivity (Kalappa et al., 2015), rescued current loss. Our findings highlight the importance of PIP_2_ interaction in causation and potential therapy for *KCNQ2* encephalopathy.

## Materials and Methods

### Ethics

Animal procedures were approved by the Institutional Animal Care and Use Committee (IACUC) at Baylor College of Medicine, in accordance with guidelines of the Association for Assessment and Accreditation of Laboratory and Animal Care (AAALAC) and the U.S. National Institutes of Health (NIH). Clinical data were collected according to procedures reviewed and approved by the Institutional Review Board of Baylor College of Medicine. The study protocol allows inclusion of information on registered patients enrolled after informed written consent, as well as deidentified data regarding anonymous patients entered after verbal parental consent to the treating physician.

### cDNA Constructs, Drugs, and Nomenclature

Human KCNQ2 (Genbank: NM_004518) and KCNQ3 (NM_004519) cDNA (Biervert et al., 1998; Schroeder et al., 1998) clones were provided by Thomas Jentsch and subcloned into pcDNA3.1 (Thermo Fisher Scientific). Point mutations were introduced using QuikChange (Agilent Technologies). *C. intestinalis* voltage sensitive phosphatase (ciVSP) cDNA (Murata and Okamura, 2007) was provided by Yasushi Okamura (Osaka Univ.). Mouse phosphatidylinositol-4-phosphate 5-kinase type I gamma (PIP5K, NM_001293647) cDNA was provided by Robin Irvine (Cambridge Univ.). Human M1 muscarinic receptor (M1R, NM_000738.3) cDNA was provided by Bruce Conklin (University of California, San Francisco). SF0034 was provided by SciFluor Life Sciences (Cambridge, MA). Oxotremorine methiodide (Oxo-M) was purchased from (Sigma-Aldrich). Kv7.2 protein variants are listed with respect to the 872 codon reference sequence (NCBI Reference Sequence: NM 172107.3).

### Cell Culture and cDNA Expression

Chinese hamster ovary (CHO) cells and human embryonic kidney (HEK) 293 T cells were maintained in Dulbecco’s modified Eagle’s medium (DMEM, Gibco) at 5% CO_2_ and 37°C. All patch clamp studies shown were conducted using CHO cells transfected using PolyJet (SignaGen). To achieve higher protein expression levels required for cycloheximide (CHX) protein stability experiments, HEK cells transfected using Lipofectamine 2000 (Thermo Fisher Scientific) were used. Electroporation using the Neon system (Thermo Fisher Scientific) was used with CHO cells for surface protein biotinylation experiments and CaM co-immunoprecipitation, and with acutely dissociated hippocampal neurons for AIS localization experiments.

### Whole Cell Electrophysiology

Transfected CHO cells were recorded at room temperature (20–22°C), 1–3 days post-transfection, using an Axopatch 200B (Molecular Devices) amplifier, pCLAMP v.9, a cFlow perfusion controller and mPre8 manifold (Cell MicroControls), and glass micropipettes (VWR International) with 1–4 MΩ resistance. The extracellular solution consisted of (in mM): 138 NaCl, 5.4 KCl, 2 CaCl_2_, 1 MgCl_2_, 10 glucose, 10 HEPES, pH 7.4 with NaOH (Miceli et al., 2013). Pipette solution contained (in mM): 140 KCl, 2 MgCl_2_, 10 EGTA, 10 HEPES, and 5 Mg-ATP, pH 7.4 with KOH. Series resistance was compensated by 70% after compensation using Axopatch 200B fast and slow capacitance controls. Currents were digitally sampled at 5–10 kHz, depending on the protocol, and filtered at 5 kHz using a low-pass Bessel filter. For voltage-activation experiments, cells were held at −80 mV and depolarized in 10 mV incremental steps from −80 to +40 mV for 1 s, then stepped to 0 mV for 60 ms. Tail currents at 0 mV were fitted using the Boltzmann function: $\frac{G}{Gmax}=\frac{1}{1+e^{\frac{V_{\frac{1}{2}}-Vm}{k}}}$ to obtain the half-maximum activation voltage (*V_1/2_*) and the slope factor (*k*). Activation and deactivation rates were measured in SigmaPlot or Clampfit using a single exponential function: *I* = *Ae^−t/τ^* + *C*. TEA sensitivity experiments were performed as previously described (Soldovieri et al., 2016). Currents were elicited by 3 s ramp depolarizations from −80 to 40 mV. To monitor inhibition by Oxo-M, cells were pulsed every 2 s for 200 ms to +40 mV from a holding potential of −80 mV before, during and after perfusion with drug solution. In experiments using ciVSP to study recovery, cells were held at −80 mV, then pre-pulsed for 2 s at −20 mV before stepping to +100 mV for 10 s, and then held at −20 mV for 30 s to monitor recovery. Kinetic parameters for current depletion and recovery were determined by non-linear regression models in SigmaPlot. Rates of current decline after ciVSP activation were estimated by fitting to a sigmoidal equation: $y=\frac{1}{\left(1+e^{-t-t0/b}\right)}$. For WT Kv7.2 + Kv7.3 heteromers, current recovery kinetics after the end of a ciVSP-activating step were fit using the squared exponential function: $y^{2}=\left(1–2e^{-\frac{t}{\tau }}+e^{-2t/\tau }\right)$ as described previously (Falkenburger et al., 2010; Alberdi et al., 2015). Current recovery for channels containing variants were fit poorly using this squared exponential but could be better fit using another sigmoidal equation, the 4-parameter logistic: $y=ymax+(y0-\frac{ymax}{(1+\frac{t}{{\left(ymax-\frac{ymin}{2}\right)}^{b}}}$). A calculated liquid junction potential (−3.62 mV) was not corrected.

### Biotinylation, Immunoprecipitation, and Western Blotting

CHO cell membrane protein biotinylation was performed 48 h after electroporation, using Sulfo-NHS-LC-Biotin (Pierce) as described previously (Kosenko et al., 2012). Excess biotin was quenched using 100 mM glycine and cells were lysed. After clearing by centrifugation, NeutrAvidin agarose beads (Pierce) were used to collect biotinylated proteins. For CaM co-IP, lysates were incubated with Dynabead protein A magnetic beads (Thermo Fisher) pre-coated with guinea pig anti-KCNQ2-C2 antibodies (Surti et al., 2005). Proteins were separated on 8% (Kv7) or 12% (CaM) SDS-PAGE gels, transferred onto nitrocellulose membranes, and analyzed by western blotting. For CaM blots, PVDF membranes were used, and protein gels were transferred in 0.1 M potassium phosphate buffer (pH 7.4) overnight and then fixed in 0.2% glutaraldehyde for 45 min. Membranes were blocked and then blotted with antibodies, imaged using enhance chemiluminescence (ThermoFisher Pierce Supersignal) and film, and bands were quantified as described previously (Cooper et al., 2001; Pan et al., 2006; Jin et al., 2009). Primary blotting antibodies were guinea pig-anti-KCNQ2-C2 and anti-KCNQ3-N1, mouse-anti-calmodulin (Pierce), and chicken-anti-GAPDH as loading control (Cell Signal) in 2% IgG-Free BSA (Jackson ImmunoResearch), blocking buffer was tris-buffered saline with 0.1% Tween-20 (TBS-T, Bio-Rad), and secondary antibodies were HRP-conjugated anti-guinea pig, anti-mouse or anti-chicken (Jackson ImmunoResearch). For quantification of channel or calmodulin expression in whole cell lysates, each channel band was normalized against the intensity of the anti-GAPDH band in the same lane. Bar graphs show results of normalized versus WT control samples. Band intensities were averaged across the indicated numbers of independent transfections and blots.

### Cycloheximide Treatment

Forty-eight hours after transfection, HEK cells were treated with 100 μg/ml of CHX (Sigma-Aldrich) or dimethyl sulfoxide (DMSO) (Thermo Fisher) at the same start time and then processed for cell lysis at subsequent time points as shown.

### Hippocampal Neuron Culture and Immunocytochemistry

Hippocampi from male and female Sprague-Dawley rats (Charles River) were dissected at E18–19 incubated with proteases at 37° C for 15 min, and cells were dissociated by trituration with fire-polished Pasteur pipettes. After electroporation (Thermo Fisher Neon, 1 μg total DNA per sample, 1,200 V, 40 ms, 1 pulse), neurons were plated on poly-D-lysine (Sigma-Aldrich) and rat tail collagen (Roche) coated 12 mm glass coverslips (Warner Instruments) at 5 × 10^4^ cells per well in a 24-well plate in a plating media [minimal essential medium (MEM), 10% FBS, 0.5% glucose, 1 mM sodium pyruvate, 25 μM glutamine, and 50 units penicillin/streptomycin]. After 4 h, medium was changed to Neurobasal medium supplemented with B-27 and 25 mM glutamine and 50 units pen/strep. At 3 days *in vitro* (DIV), neuronal cultures were treated with 2 μM cytosine arabinoside. Half of the neuronal culture medium was replaced with fresh medium every 5–6 days. At 7 DIV, the neurons were fixed with 1% PFA in Na-acetate, pH 6.0 for 20 min and washed with PBS. Coverslips were blocked in 3% BSA, 0.1% Triton X-100 for 1 h at RT and then incubated with lab-made primary rabbit-anti-KCNQ2-N1 and guinea pig-anti-KCNQ3-N1 antibodies as previously described (Cooper et al., 2001; Devaux et al., 2004; Pan et al., 2006). Labeling with mouse-anti-Ankyrin-G (clone N106/36; UC Davis/NINDS/NIMH NeuroMab Facility) was used to demark AISs. Secondary antibodies were anti-rabbit DyLight 488, anti-mouse Cy3, and anti-guinea-pig Cy5-conjugated secondary antibodies (Jackson ImmunoResearch). Cover slips were mounted in ProLong Gold with DAPI (Thermo Fisher). Images were acquired using a Nikon 80i microscope, a 60× NA 1.4 Plan Apo oil-immersion objective lens, NIS-Elements software (Nikon), and an ExiAqua (QImaging) CCD camera. In each experiment, acquisition settings for each color channel were identical for all samples. Quantification of the immunofluorescence signals along the AIS was performed as described previously (Battefeld et al., 2014). The NIS-Elements intensity profile tool was used to mark the AIS trajectory, using abrupt increases and decreases in labeling for ankyrin-G to mark the AIS origin and distal tip. For each color channel in each set of images acquired in parallel, the black (0 intensity) level was specified by measuring a minimal intensity value at a non-AIS location; this quantity was subtracted from all measurements for all images to be compared. Intensity values along each AIS trajectory were binned in 16 segments, and these values were averaged to determine mean segment intensities for each condition and fluorophore. These values were normalized to the mean maximal values measured in images of control neurons electroporated with WT Kv7.2 and Kv7.3 cDNAs to obtain relative intensity values at each distance along the AIS (Battefeld et al., 2014).

### Statistics

Statistical analyses were performed in SigmaPlot (v.13). One‐ or two-way ANOVA was followed by Tukey’s or Bonferroni’s post-hoc test for multiple comparisons. For drug treatment studies, one‐ or two-way repeated measures (RM) ANOVA was performed. On figure panels: ^*^p < 0.05, ^**^p < 0.01 and ^***^p < 0.001. All fluorescence intensities, band intensities, and electrophysiology analyses are reported as mean ± SEM.

## Results

### Clinical Descriptions

Description of patients with EIEE and the *KCNQ2 de novo* variants c.1009G>A; p.Ala337Thr (Millichap et al., 2016) and c.1010C>G; p.Ala337Gly (Saitsu et al., 2012) appeared previously. The A337T individual is now age 8 years of age and has been followed clinically since birth. Electroencephalograms (EEGs) at ages 6 days, 5 months (prior to ezogabine use), and 6 months (with high dose ezogabine) were presented previously. Focal tonic seizures and clusters of flexor spasms, hypotonia, and encephalopathy were noted at age 2 days. The neonatal EEG background had primarily a burst-suppression appearance, except for brief intervals of continuity (high-amplitude delta activity with multifocal epileptiform discharges) during wakefulness. The EEG evolved by age 4 months to a modified hypsarrhythmia pattern with multifocal epileptiform activity and frequent electroclinical focal tonic seizures (several per day) continued despite serial anti-seizure medication trials including phenobarbital, topiramate, oxcarbazepine, and lamotrigine. Ezogabine treatment at age 5 months was followed closely with serum levels, EEG, and clinical observation. Ezogabine caused urinary retention at 23 mg/kg/day but this resolved easily and the treatment was resumed at 14 mg/kg/day and later increased to 21 mg/kg/day. Ezogabine exposure was associated with improvement in seizures, which was reflected in diminished epileptiform activity. The patient remained on ezogabine until it was removed from the market by the manufacturer almost 4.5 years later. Despite ezogabine, however, the patient remains globally developmentally delayed (nonverbal but can choose objects by pressing a button on an assistive device, non-ambulatory), although breakthrough seizures became rare. The A337G individual was described at 7 years old in 2012 (Saitsu et al., 2012). Very briefly, the patient was diagnosed with Ohtahara syndrome as a neonate, due to tonic seizure onset at age 7 days, burst-suppression EEG, and encephalopathy. The patient remained globally impaired at age 7 years, without the ability to speak meaningful words or walk.

### Kv7.2 A337T and A337G Are Loss-of-Function Variants

Under whole cell voltage-clamp, CHO cells transfected with WT Kv7.2 produced large, slowly activating, non-inactivating currents, but cells expressing A337T alone or A337G alone gave little detectable voltage-activated current (Figures 1B,C). Compared to WT Kv7.2, CHO cells transfected with A337T + Kv7.2 plasmids in a 1:1 ratio to mimic the heterozygous genotype displayed about 40% less current at +40 mV and A337G + Kv7.2 about 80% less current (Figure 1C; Kv7.2: 89.6 ± 9.6 pA/pF; A337G + Kv7.2: 20.2 ± 3.6 pA/pF; A337T + Kv7.2: 55.3 ± 11.9 pA/pF). The conductance/voltage relationship of cells co-expressing WT and A337 variants 1:1 was not significantly changed compared to WT alone (Figure 1D). Experiments using co-expression with the tetraethylammonium (TEA)-resistant mutant, Kv7.2 Y284C (Hadley et al., 2003; Soldovieri et al., 2016), were used to assess for co-assembly. The principle of this assay is that channels formed by coassembly with Y284C exhibit an intermediate sensitivity to TEA. A337T and A337G, when co-expressed with Y284C (or with WT), indeed showed currents that were more sensitive to TEA than Y284C expressed alone (Supplementary Figure S1). Thus, the A337 variants are capable of forming tetramers with Y284C, and the most parsimonious explanation of the results is that in homomers, A337T partly and A337G more fully exerts a current-suppressing effect on WT Kv7.2.

We used surface biotinylation and western blots to further assess whether the observed decreases in current could be explained by a reduction of protein levels. Total and surface-localized Kv7.2 protein levels from cells expressing A337G were not significantly different from WT controls. Cells expressing A337T alone showed significantly decreased total Kv7.2 protein (0.56 ± 0.08 of WT) and reduced surface abundance (0.43 ± 0.06 of WT). These reductions were insufficient to account for the nearly complete loss of current exhibited (Figure 1E). Moreover, when either A337T or A337G was co-expressed with WT Kv7.2 in a 1:1 ratio, total protein (Kv7.2: 1.00 ± 0.11; A337G + Kv7.2: 0.78 ± 0.17; A337T + Kv7.2: 0.73 ± 0.17) and surface abundance (Kv7.2: 1.00 ± 0.01; A337G + Kv7.2: 1.23 ± 0.12; A337T + Kv7.2: 1.20 ± 0.16) were restored to control levels (Figure 1F; see Supplementary Figure S2 for full blots).

**Figure 2:**
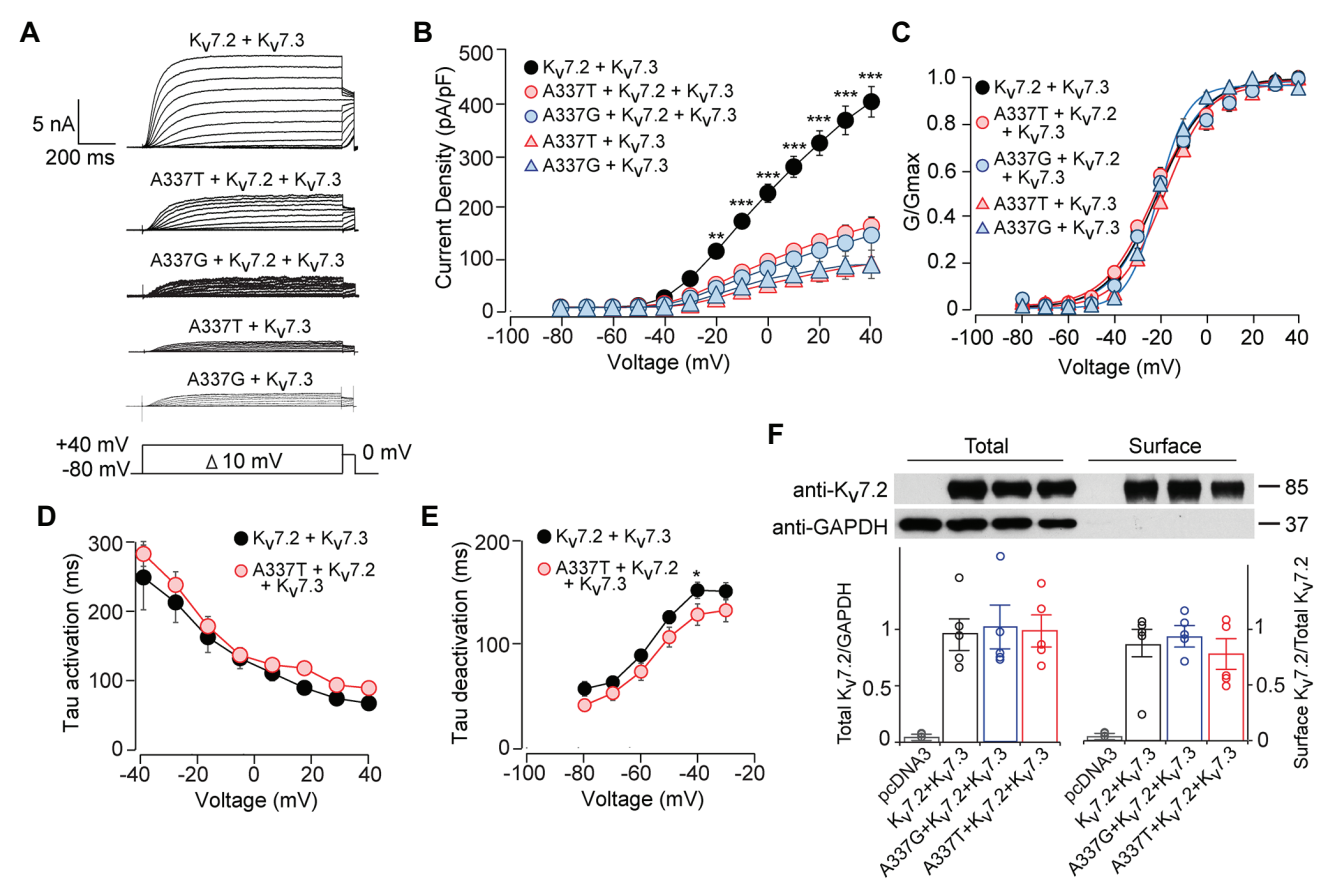
A337T and A337G strongly suppressed heteromeric Kv7.2 + 7.3 currents. **(A)** Currents produced by the indicated combinations of subunits; voltage protocol is shown as an inset. **(B)** Current-voltage plots show significant reductions by A337T and A337G compared to control at voltages of −20 and above (^**^p < 0.01, ^***^p < 0.001, Two-way ANOVA, Bonferroni test, *n* = 10–20, see Table 1). **(C)** Conductance-voltage plots of WT only and mutant-containing channels (*n* = 5–20, ^*^p = 0.02, ^***^p < 0.001, Two-way ANOVA, Bonferroni test; see Table 1). **(D,E)** Single exponential fits for heteromeric channels showing the **(D)** activation time constant and **(E)** deactivation time constant (at −40 mV, ^*^p < 0.05, Two-way ANOVA, Bonferroni test, *n* = 6–10). **(F)** Western blot analysis of cell lysates (left lanes) and isolated biotinylated membrane surface proteins (right lanes) from CHO cells expressing Kv7.2 + Kv7.3 co-transfected with A337 variants (*n* = 5). Upper, representative results; lower, quantification performed as for Figure 1. Images are of the entire immunoblots are shown as supplementary information (Supplementary Figure S3).

Although homomeric Kv7.2 channels are found within the nervous system, Kv7.2 + Kv7.3 heteromeric channels give much larger currents and appear to be the main contributors to neuronal somatic and axonal M-currents in many neuronal types (Wang et al., 1998; Cooper et al., 2000; Hadley et al., 2003; Devaux et al., 2004; Pan et al., 2006; Battefeld et al., 2014). Therefore, we co-transfected each A337 variant with WT Kv7.2 and Kv7.3 cDNAs in a 1:1:2 ratio, mimicking the heterozygous genotype and the heteromeric subunit composition. Remarkably, inclusion of either A337T or A337G as 25% of total cDNA suppressed maximal current density by about 60% (Figures 2A,B: at +40 mV, Kv7.2 + Kv7.3: 416.0 ± 30.9 pA/pF; A337T + Kv7.2 + Kv7.3: 164.7 ± 18.6 pA/pF; A337G + Kv7.2 + Kv7.3: 146.4 ± 30.3 pA/pF). Transfection with a 1:1 ratio of A337T or A337G and Kv7.3 cDNA produced current density levels that were decreased by nearly 80% compared to WT alone (at +40 mV, A337T + Kv7.3: 88.5 ± 14.3 pA/pF; A337G + Kv7.3: 87.8 ± 28.7 pA/pF). No differences were observed between the conductance-voltage curves of Kv7.2 + Kv7.3, A337T + Kv7.2 + Kv7.3, and A337G + Kv7.2 + Kv7.3, but a small rightward shift in V_1/2_ was observed for A337T + Kv7.3, and the calculated steepness of voltage activation was slightly increased for both A337T + Kv7.3 and A337G + Kv7.3 (Figure 2C; Table 1). In addition, activation and deactivation rates were not changed in A337T + Kv7.2 + Kv7.3 channels compared to WT heteromers (Figures 2D,E). Neither variant reduced total or surface biotinylated Kv7.2 protein levels (Figure 2F; see Supplementary Figure S3 for full blots).

**Table 1:** Current density and activation gating parameters of wild type or variant-containing Kv7.2 and Kv7.2/7.3 channels.

Transfection	*n*	Current density pA/pF, +40 mV	*p*	V_1/2_ (mV)	*p*	*k*, fold (mV/e)	*p*
Kv7.2	13	89.6 ± 9.6		−21.7 ± 1.0		11.7 ± 0.5	
A337G	6	6.5 ± 1.1	<0.001^1^	n/a		n/a	
A337T	10	4.9 ± 1.6	<0.001^1^	n/a		n/a	
Kv7.2 + A337G	11	20.2 ± 3.6	<0.001^1^	−24.1 ± 1.2		12.1 ± 1.9	NS
Kv7.2 + A337T	15	55.3 ± 11.9	0.001^1^	−23.5 ± 0.8		12.7 ± 1.0	NS
Kv7.2 + Kv7.3	20	416.0 ± 30.9		−21.5 ± 0.8		10.6 ± 0.4	
Kv7.2 + Kv7.3 + A337G	9	146.4 ± 30.3	<0.001^2^	−21.7 ± 0.9		0.04 ± 1.0	NS
Kv7.2 + Kv7.3 + A337T	17	164.7 ± 18.6	<0.001^2^	−23.3 ± 1.2		11.7 ± 0.7	NS
Kv7.3 + A337G	6	87.8 ± 28.7	<0.001^2^	−23.7 ± 0.4		6.6 ± 0.5	<0.001^2^
Kv7.3 + A337T	12	88.5 ± 14.3	<0.001^2^	−18.0 ± 0.4		9.8 ± 0.5	<0.001^2^
Kv7.2 + PIP5K	10	238.8 ± 50.0	<0.001^3^	−39.6 ± 1.38	<0.001^3^	8.5 ± 0.6	<0.001^3^
A337G + PIP5K	10	48.4 ± 24.6	<0.001^3^ <0.001^4^	n/a		n/a	
A337T + PIP5K	9	47.5 ± 15.9	<0.001^3^ <0.001^4^	n/a		n/a	
Kv7.2 + A337G + PIP5K	9	199.0 ± 35.4	<0.001^3^ NS^4^	−34.8 ± 0.8	<0.001^3^ NS^4^	9.6 ± 0.9	NS
Kv7.2 + A337T + PIP5K	9	162.6 ± 47.9	<0.001^3^ NS^4^	−39.0 ± 0.5	<0.001^3^ NS^4^	10.0 ± 0.6	NS
Kv7.2 + Kv7.3 + PIP5K	8	643.0 ± 58.9	<0.001^3^	−33.5 ± 0.6	<0.001^3^	9.6 ± 0.7	NS
Kv7.2 + Kv7.3 + A337G + PIP5K	9	537.4 ± 46.9	<0.001^3^ NS^5^	−33.5 ± 0.5	<0.001^3^ NS^5^	9.2 ± 0.6	NS
Kv7.2 + Kv7.3 + A337T + PIP5K	9	725.4 ± 45.5	<0.001^3^ NS^5^	−33.0 ± 0.5	<0.001^3^ NS^5^	9.0 ± 0.6	NS

1 vs. WT Kv7.2 alone.

2 vs. WT Kv7.2 + Kv7.3 alone.

3 vs. same subunits, without PIP5K.

4 vs. Kv7.2 + PIP5K.

5 vs. Kv7.2 + Kv7.3 + PIP5K.

To analyze the losses of total and membrane protein when A337T was expressed alone, we compared the protein stability of WT Kv7.2 and A337T channels in transfected HEK cells using the translation inhibitor, cycloheximide. After CHX treatment, A337T protein degradation was more rapid than WT (Supplementary Figure S4). Treatment with 0.1% DMSO (the CHX vehicle) gave no significant effects over 24 h, but A337T protein levels were lower than the WT, as shown above using CHO cells (see Supplementary Figure S5 for full blots).

**Figure 3:**
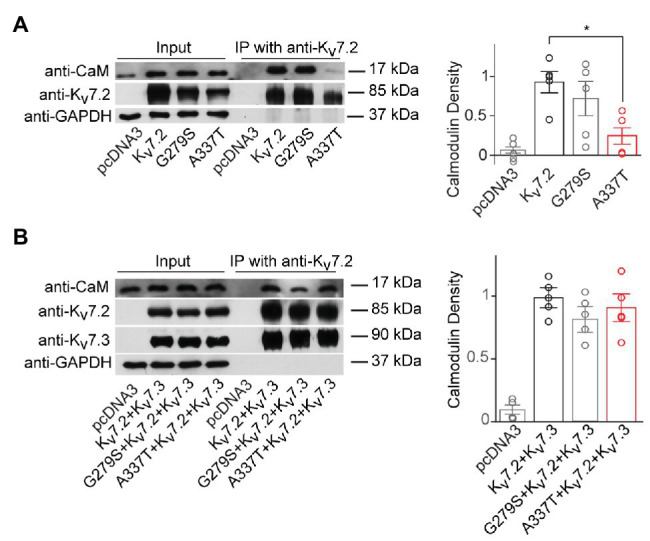
A337T homomers are less able to co-immunoprecipitate calmodulin than WT Kv7.2 homomers. **(A)** Representative western blot analysis and quantification of calmodulin (CaM) co-immunoprecipitated using anti-Kv7.2 antibodies and transfected human embryonic kidney (HEK) cells expressing WT Kv7.2, G279S, or A337T. CaM co-immunoprecipitated is significantly reduced for A337T (^*^p = 0.04, One-way ANOVA, Tukey test, *n* = 5) but not G279S. In plots, CaM immunoprecipitated is normalized to wild type (WT). **(B)** Representative western blot analysis and quantification of CaM co-immunoprecipitated from cells expressing WT Kv7.2 + Kv7.3, alone or in a 1:1:2 ratio with G279S or A337T, show no differences between groups (*n* = 4). These cropped blot images are from full-length gels shown as supplementary information (Supplementary Figure S6).

Next, we used co-immunoprecipitation to compare effects of A337T on the binding of CaM by homomers and heteromers. Expressed alone, A337T exhibited significantly decreased ability to co-precipitate CaM, whereas binding by G279S, a missense change at the GYG selectivity filter with strongly dominant-negative effects on conduction (Schroeder et al., 1998), was similar to WT (Kv7.2: 1.00 ± 0.16; G279S: 0.71 ± 0.22; A337T: 0.24 ± 0.12; Figure 3A). However, in heteromeric channels containing the A337T variant subunit, CaM-bound levels were comparable to WT and G279S controls (Figure 3B). Thus, for A337T, reduced protein levels were correlated with reduced half-life and CaM binding. However, the near-absence of current observed when A337T or A337G were expressed alone and the partial loss of current seen when the mutant subunits were co-expressed to mimic heterozygosity could not be explained by these mechanisms (see Supplementary Figure S6 for full blots).

### In Cultured Hippocampal Neurons, Three Pore Domain Variants (A274M, A294V, and G279S) Reduce AIS Trafficking, but A337T Localizes Normally to the AIS

**Figure 4:**
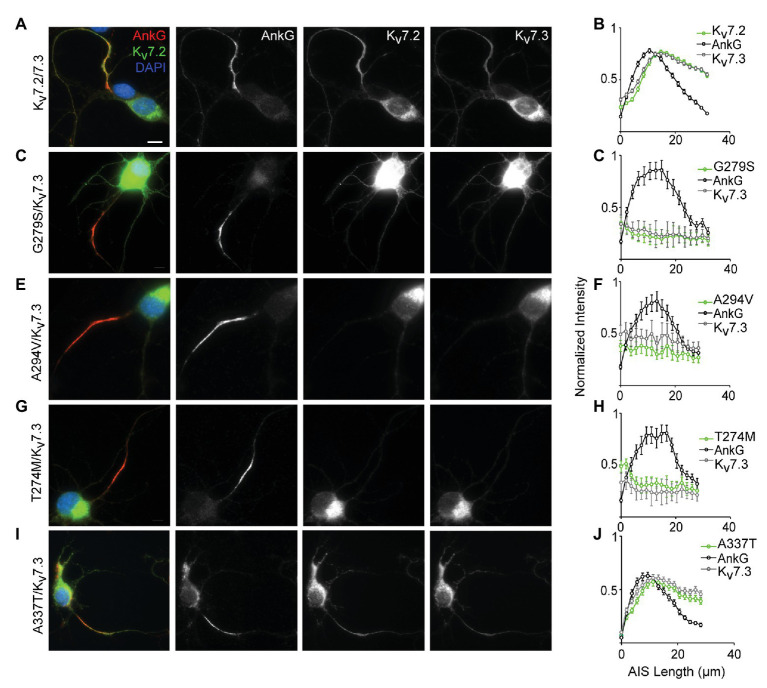
A337T does not disrupt localization of Kv7.2 or Kv7.3 subunits at the AIS of hippocampal neurons. **(A)** Representative image of cultured rat hippocampal neurons electroporated with WT Kv7.2 + Kv7.3 cDNAs at E18 and imaged after 7 DIV. Neurons were labeled with DAPI, anti-AnkG, anti-Kv7.2, and anti-Kv7.3. Merged (color) and greyscale images show Kv7.2 + Kv7.3 localized at the AIS (scale, for all images: 10 μm). **(B)** Intensity profile quantifications of AnkG, Kv7.2, and Kv7.3 antibody labeling along the AIS. **(C–H)** Representative images of hippocampal neurons electroporated with pore mutations G279S, A294V, and T274M and their respective intensity profile quantifications. Expression of these variants prevents concentration of Kv7.2 and Kv7.3 at the AIS. **(I,J)** Representative image of A337T and its intensity profile quantification show a normal pattern of Kv7.2 and Kv7.3 localization at the AIS (histograms: *n* = 69–104 neurons).

Localization of Kv7.2/Kv7.3 heteromeric channels at the AIS is critical for their roles in regulating neuronal excitability (Battefeld et al., 2014). To assess for potential AIS trafficking effects of variants, we electroporated cDNAs into freshly dissociated hippocampal neurons at embryonic day 18–19 (E18–19) and analyzed their ability to become concentrated along the AIS as axons develop in culture. At 7 DIV, ankyrin-G is highly concentrated at the AIS but endogenous Kv7.2 and Kv7.3 remain weakly expressed and are undetectable at the AIS under our imaging conditions (Nakada et al., 2003; Rasmussen et al., 2007; Abidi et al., 2015). To maximize sensitivity for observing an effect of the pathogenic Kv7.2 variants, each was co-expressed with Kv7.3 in a 1:1 ratio, i.e., a subunit composition that produced 80% current loss in CHO cells. Neurons co-expressing WT Kv7.2 and Kv7.3 exhibited concentrated staining for both subunits in the AIS (Figure 4A). Quantification of the relative staining intensity along the AIS revealed a length profile similar to that seen *in vivo* (Battefeld et al., 2014), with light labeling proximally, and strong labeling in the middle and distal AIS (Figure 4B). By contrast, three previously studied pore domain variants, G279S, A294V, and T274M (Peters et al., 2005; Orhan et al., 2014; Abidi et al., 2015), did not concentrate at any portion of the AIS and were instead retained at the soma (Figures 4C–H). Unlike these pore variants, A337T proteins were concentrated in the AIS in a pattern that was similar to that seen in neurons co-expressing Kv7.2 and Kv7.3 only (Figures 4I,J). Thus, even as homozygous heteromers, A337T-containing channels traffic to the AIS similarly to WT Kv7.2 + Kv7.3 heteromers, while the three different pore domain variants appear to strongly suppress AIS trafficking.

### A337T and A337G Alter Channel Regulation by PIP_2_

**Figure 5:**
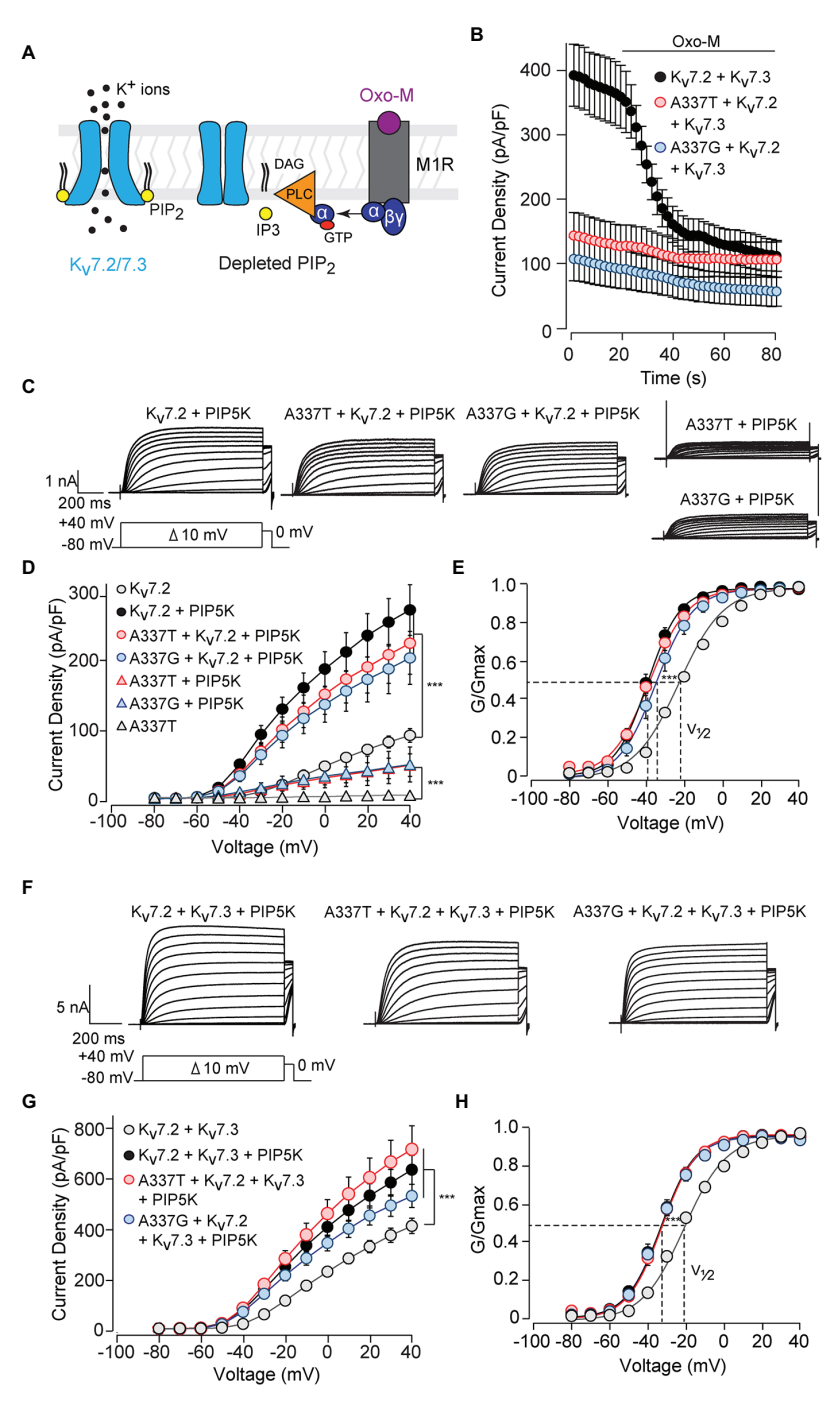
Currents suppressed by A337 variants were restored by co-expression of PIP5K. **(A)** Diagram of the main transduction pathway underlying cholinergic inhibition of M-current. Agonist oxotremorine methiodide (Oxo-M) activates the M1 muscarinic receptor (M1R), activating a heterotrimeric G-protein (α, βγ) and phospholipase C (PLC). PLC cleaves phosphatidylinositol 4,5-bisphosphate (PIP_2_) residing in the membrane, reducing PIP_2_ available for binding to the channel. **(B)** Time course of current inhibition by Oxo-M (10 μM, bar) in CHO cells transfected with M1R, Kv7.2, and Kv7.3, with and without A337 variants. **(C)** Representative families of currents produced in response to step depolarizations by the indicated combinations of WT and mutant Kv7.2 subunits when co-transfected with PIP5K. **(D)** Current-voltage relationships for channels co-expressed with PIP5K compared to without PIP5K (^***^p < 0.001, two-way ANOVA, Bonferroni test, *n* = 5–16, see Table 1). **(E)** Conductance-voltage relationships from cells transfected with PIP5K compared to WT Kv7.2 without PIP5K. Activation of WT Kv7.2 expressed alone, WT + A337T, and WT + A337G are significantly shifted to hyperpolarized potentials (^***^p < 0.001, two-way ANOVA, Bonferroni test, *n* = 5–16, see Table 1). **(F)** Representative currents produced by the indicated combinations of heteromeric WT and mutant Kv7.2 subunits in cells co-transfected with PIP5K. **(G)** Current densities in cells co-expressing PIP5K compared to WT Kv7.2 + Kv7.3 (^***^p < 0.001, Two-way ANOVA, Bonferroni test, *n* = 10–20). **(H)** Conductance-voltage relationships from cells transfected with PIP5K compared to WT Kv7.2 + Kv7.3 expressed without PIP5K. PIP5K overexpression shifts activation significantly to hyperpolarized potentials (^***^p < 0.001, two-way ANOVA, Bonferroni test, *n* = 10–20, see Table 1).

Activation of Gq-coupled receptors, including the M1 muscarinic acetylcholine receptor, rapidly inhibits neuronal Kv7 currents (Brown and Adams, 1980; Delmas and Brown, 2005). The primary signaling pathway for this inhibition is depletion of membrane PIP_2_, leading to dissociation of PIP_2_ from channel binding sites and uncoupling of voltage sensing from opening of the ion pore (Figure 5A; Suh and Hille, 2002; Zhang et al., 2003; Falkenburger et al., 2010; Zaydman et al., 2013). Given the obligatory role of PIP_2_ in Kv7 activation, we investigated whether these variants affected PIP_2_ interaction. Treatment of CHO cells co-expressing M1 receptors and WT Kv7.2 + Kv7.3 with the muscarinic agonist oxotremorine methiodide (Oxo-M) strongly inhibited currents (Figure 5B; pre-Oxo-M, 391.5 ± 48.5 pA/pF; after 20 s Oxo-M, 158.5 ± 28.0 pA/pF, 41 ± 7% of pre). As expected, channels formed from 1:1:2 co-expression of WT Kv7.2, an A337 variant, and Kv7.3, gave much smaller basal currents. However, these small currents were also, proportionally, less completely suppressed by Oxo-M (A337T: pre-Oxo-M, 140.9 ± 36.4 pA/pF; after 20 s Oxo-M, 107.8 ± 29.3 pA/pF, 77 ± 20% of pre; A337G: pre-Oxo-M, 104.4 ± 36.3 pA/pF; after 20 s Oxo-M, 71.7 ± 28.6 pA/pF, 69 ± 27% of pre).

The pattern of smaller basal currents and lessened suppression by Oxo-M resulting from co-expressing the variants could reflect reduced ability to be activated by depolarization, despite binding of PIP_2_. To further assess this, we used co-expression of the PIP_2_-synthesizing enzyme PIP5K to boost the bulk membrane PIP_2_ concentration (Falkenburger et al., 2010; Kim et al., 2016b; Soldovieri et al., 2016). In cells expressing WT Kv7.2 subunits alone, PIP5K overexpression increased current density nearly 3-fold (Figures 5C,D) and shifted the conductance-voltage curve to the left (Figure 5E). Remarkably, co-expressing PIP5K with a 1:1 ratio of one variant and WT Kv7.2 subunits augmented currents strongly. Indeed, current densities were not significantly different from WT alone (At +40 mV, Kv7.2: 89.6 ± 9.6 pA/pF; Kv7.2 + PIP5K: 266.8 ± 35.8 pA/pF; A337T + Kv7.2 + PIP5K: 219.8 ± 43.7 pA/pF; A337G + Kv7.2 + PIP5K: 199.0 ± 37.3 pA/pF; Figure 5D; Table 1). Also, whereas expression of A337T or A337G alone gave current barely detectable above background, with PIP5K overexpression, A337T or A337G currents were readily detected (Figure 5D). Indeed, for A337T or A337G with PIP5K, current density at +40 mV was about half of WT without exogenous PIP5K (A337T: 4.9 ± 1.6 pA/pF; A337T + PIP5K: 47.5 ± 15.9 pA/pF; A337G: 6.5 ± 1.1 pA/pF; A337G + PIP5K: 48.4 ± 24.7 pA/pF; Figure 5D).

Next, we repeated the PIP5K co-expression experiment for WT Kv7.2 + Kv7.3 heteromers with and without A337T or A337G. Whereas currents in this configuration were diminished strongly by the co-expression of the A337 variants (Figures 2A,B), PIP5K increased current in cells expressing either variant in 1:1:2 ratio with WT Kv7.2 and Kv7.3 to levels equal to WT (at +40 mV, Kv7.2 + Kv7.3: 416.0 ± 30.9 pA/pF; Kv7.2 + Kv7.3 + PIP5K: 643.0 ± 58.3 pA/pF; A337T + Kv7.2 + Kv7.3 + PIP5K: 725.4 ± 95.5 pA/pF; A337G + Kv7.2 + Kv7.3 + PIP5K: 537.4 ± 46.9 pA/pF, Figures 5F,G; see Table 1). The conductance-voltage curves of WT and A337T or A337G-containing heteromers were left-shifted equally (Figure 5H). In contrast, channels formed by 1:1 co-expression of Kv7.2 and G279S, a mutation within the selectivity filter that exhibits dominant-negative effects (Schroeder et al., 1998), were only partly restored in amplitude by overexpressed PIP5K (Supplementary Figure S7). In combination, the M1 receptor and PIP5K experiments indicated that including A337T or A337G subunits within channel tetramer confers reduced PIP_2_ responsiveness upon channels formed by these subunits and WT Kv7.2 or Kv7.2 and Kv7.3. Furthermore, these effects can be reversed, all or in part, by raising the cellular PIP_2_ concentration with PIP5K overexpression. In contrast to channels incorporating G279S variants, A337T and A337G containing channels appeared able to conduct well and to couple voltage-sensing and pore opening well, but to require higher levels of PIP_2._

**Figure 6:**
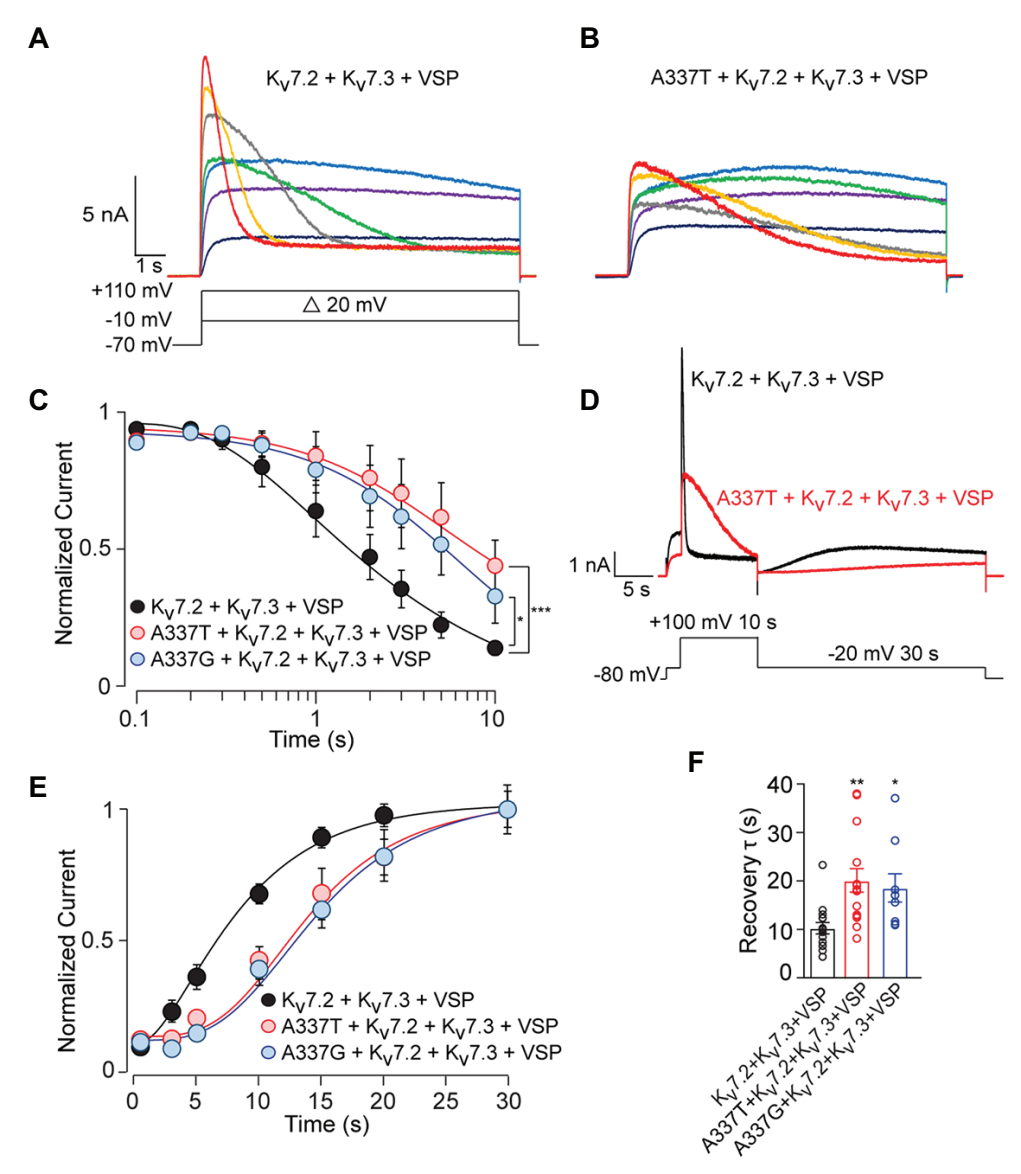
*C. intestinalis* voltage sensitive phosphatase (ciVSP) induced current suppression and recovery is slower in cells expressing A337T or A337G. **(A,B)** Currents produced by Kv7.2 + Kv7.3 **(A)** or A337T + Kv7.2 + Kv7.3 **(B)**, in response to 10 s step depolarizations, in cells co-expressing ci-VSP. The voltage protocol in A applies. **(C)** Normalized current densities from cells co-transfected with the indicated subunits and ciVSP, at intervals during 10 s depolarizing steps to +100 mV (At 10 s, ^***^p < 0.001, ^*^p = 0.025, two-way ANOVA, Bonferroni test, *n* = 8–11). **(D)** Voltage protocol and representative currents showing ciVSP induced current inhibition (at +100 mV) and recovery at −10 mV). Both appear faster for WT Kv7.2 + Kv7.3 heteromers, compared to A337T + Kv7.2 + Kv7.3. **(E)** Recovery is delayed in cells transfected with the mutant subunits, compared to WT heteromer only controls (^***^p < 0.001, ^**^p = 0.004, two-way ANOVA, Bonferroni test, *n* = 8–11). **(F)** Recovery rates for A337T + Kv7.2 + Kv7.3, and A337G + Kv7.2 + Kv7.3 channels are significantly slowed compared to control (^*^p = 0.02, ^**^p < 0.004, one-way ANOVA, Bonferroni test, *n* = 9–15).

To analyze these mechanisms further, we co-expressed the *Ciona intestinalis* voltage-sensitive phosphatase (ciVSP) with channel subunits. Previous studies have shown that strong membrane depolarization of cells expressing ciVSP rapidly depletes membrane PIP_2_, and M-current is inhibited at rates reflecting net unbinding of PIP_2_ from the channels (Murata and Okamura, 2007; Falkenburger et al., 2010). In CHO cells co-expressing ciVSP and WT channels, 10 s long step depolarizations induced currents that activated and then declined (Figure 6A; see Supplementary Figure S8A). The onset and rate of current decline increased progressively with membrane depolarizations above +30 mV (Figure 6A). Because the M1R and PIP5K experiments indicated that A337T and A337G may reduce basal PIP_2_ binding, we expected ciVSP co-expression to reveal a hastening of unbinding for those variants, as seen in studies of R325G, a site 12 residues closer to the S6 membrane helix which may interact directly with PIP_2_ (Soldovieri et al., 2016). Unexpectedly, in cells co-expressing ciVSP and A337T + Kv7.2 + Kv7.3, at steps to +30 mV and above, the rate of current decline was slower and the fraction of current depleted after 10 s was less than for WT (Figure 6B). At +100 mV, we found that inclusion of A337T or A337G significantly delayed ciVSP-induced current declines compared to controls (Figure 6C). The decay rate constants were 3.29 s (Kv7.2 + Kv7.3), 14.80 s (A337T + Kv7.2 + Kv7.3), and 9.38 s (A337G + Kv7.2 + Kv7.3). Current decline during ciVSP activation was also slowed by co-expression of either variant with WT Kv7.2 (i.e., in homomeric channels; see Supplementary Figure S8B). It is unlikely that co-expression of A337G or A337T was slowing the rate at which ciVSP activation was depleting unbound, bulk membrane PIP_2_. Instead, inclusion of the variants seemed to slow the effective rate of dissociation from the channels (see Discussion).

Reduced equilibrium binding of a ligand to a single site can result from slowing of both “on” and “off” rates, if the change in “on” rate is greater. To investigate whether the variants very strongly slowed rates of PIP_2_ binding, we used an established depletion-recovery protocol (Falkenburger et al., 2010). PIP_2_ was extensively depleted by strong ciVSP activation (10 s at +100 mV), and then currents were monitored for 30 s at −20 mV, as PIP_2_ was resynthesized (Figure 6D). As observed previously (Falkenburger et al., 2010), recovery followed an S-shaped time course, and the rate of recovery appeared independent of the extent of prior PIP_2_ depletion (see Supplementary Figure S8C). Compared to WT channels, currents recovered more slowly in channels including the A337 variants than in WT-only channels, and this appeared due to an increase in the latency in initiation of recovery (Figures 6D–F; see Supplementary Figure S9), potentially contributing to reduced apparent affinity. However, the approximately 2-fold slowing in recovery was similar to the 3‐ to 5-fold change in the rate of current reduction seen in the depletion experiments. To further probe PIP_2_ interactions with the gating machinery, we measured activation and deactivation kinetics, since mutations near some PIP_2_ binding sites can affect the rate of deactivation (Chen et al., 2015). A337T did not influence voltage-dependent activation and deactivation rates (Figures 2D,E). Together, these data show that incorporation of A337T or A337G into heteromeric Kv7.2 + Kv7.3 channels impaired PIP_2_ interaction in a novel pattern, appearing to decrease occupancy at sites required for electromechanical coupling at basal conditions of whole cell patch recording, without hastening the unidirectional rate of unbinding if membrane PIP_2_ was strongly depleted.

### SF0034 Rescued Subthreshold Current Density in Heterozygous A337T Channels

Ezogabine, an FDA-approved drug that increases Kv7.2/7.3 currents by shifting voltage activation to more hyperpolarized voltages and increasing maximal current density, has been proposed as a targeted therapeutic in *KCNQ2* encephalopathy and use with some apparent benefit has been described in small clinical case series, including in the A337T proband (Gunthorpe et al., 2012; Millichap and Cooper, 2012; Weckhuysen et al., 2013; Millichap et al., 2016). However, ezogabine exhibits modest selectivity and potency and has side effects, including acute urinary retention and blue skin discoloration, which limit its clinical usefulness (Brickel et al., 2012; Clark et al., 2015; Food and Drug Administration, U.S., 2015; Kalappa et al., 2015). The infant with neonatal onset epileptic encephalopathy and a heterozygous A337T variant was given ezogabine at 4.5 months of age, resulting in improved seizure control and interictal electroencephalographic pattern, but urinary retention limited dosing to 21 mg/kg/day (Millichap et al., 2016), which achieves a brain concentration estimated at approximately 1 μM (Gunthorpe et al., 2012). SF0034 is an ezogabine analogue with improved potency and selectivity in *in vitro* and *in vivo* (Kalappa et al., 2015). In HEK cells, SF0034 was ~5-fold more potent than ezogabine in shifting the voltage-dependence of activation of Kv7.2 + Kv7.3 heteromers but was less efficacious than ezogabine against Kv7.4 or Kv7.5 homomers. The effects of SF0034, like for ezogabine, were abolished by point mutation of Kv7.3 Trp265. SF0034 treatment was shown to slow spike frequency in mouse CA1 pyramidal neurons, but this effect was markedly reduced in littermate mice with Kv7.2 conditionally deleted from these neurons. To begin to assess whether such “second generation” openers merit exploration as candidate therapeutic agents for *KCNQ2* encephalopathy, we studied the effects of SF0034 on channels including the A337T pathogenic variant.

**Figure 7:**
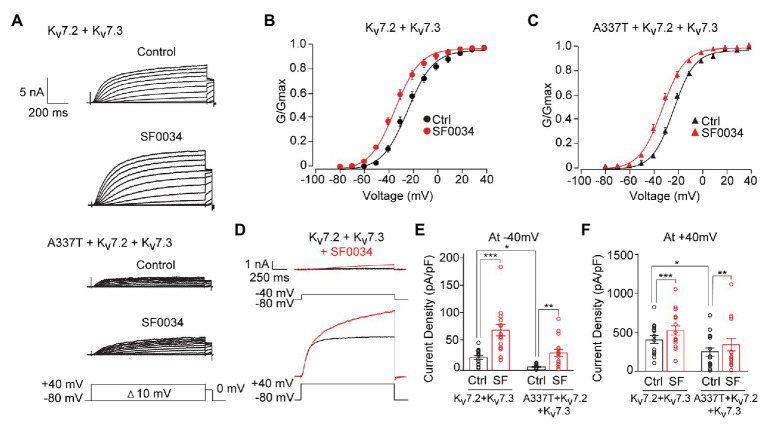
SF0034 partially restored current loss due to A337T mutation. **(A)** Kv7.2 + Kv7.3 and A337T + Kv7.2 + Kv7.3 currents in CHO cells, before and after treatment with 1 μM SF0034. Protocol given in inset. **(B)** Conductance-voltage relationship of WT Kv7.2 + Kv7.3 before and after SF0034 treatment (Control (Ctrl): V_1/2_ = −23.8 ± 0.6 mV, k = 10.7 ± 0.7 mV; SF0034: V_1/2_ = −34.8 ± 0.6 mV, k = 10.5 ± 0.7 mV (n = 16). **(C)** Conductance-voltage relationship of A337T + Kv7.2 + Kv7.3 before and after SF0034 treatment. Ctrl: V_1/2_ = 23.1 ± 0.4 mV, k = 8.9 ± 0.4 mV; SF0034: V_1/2_ = −32.5 ± 0.4 mV, k = 9.6 ± 0.5 mV (n = 16). **(D)** Kv7.2 + Kv7.3 currents response to the indicated depolarizing steps, before and after SF0034. **(E,F)** Current densities before and after SF0034 treatment for the indicated subunit combinations at −40 mV (^*^p = 0.02; ^**^p = 0.002; and ^***^p < 0.001) and +40 mV (^*^p = 0.03 and ^***^p < 0.001). SF0034 treated A337T-containing channel current density was not significantly different compared to untreated WT control at either −40 or +40 mV (two-way repeated measures ANOVA, Bonferroni test, one-way ANOVA, Kruskal-Wallis test, *n* = 16).

At 1 μM, SF0034 increased currents in cells expressing WT heteromeric channels and cells co-expressing A337T (Figure 7A). SF0034 shifted the conductance-voltage curve to hyperpolarized potentials equivalently (Kv7.2 + Kv7.3: pretreatment control V_1/2_ = −23.8 ± 0.6 mV, SF0034 V_1/2_ = −34.5 ± 0.6 mV; A337T + Kv7.2 + Kv7.3: pretreatment control V_1/2_ = −23.0 ± 0.4 mV, SF0034 V_1/2_ = −32.5 ± 0.4 mV; Figures 7B,C). Although absolute increases in current were greater with a strongly depolarizing voltage step to +40 mV compared to −40 mV (Figure 7D), relative increases were greatest at weak depolarizations (Figures 7E,F). Thus, 1 μM SF0034 increased current density of WT Kv7.2 + Kv7.3 channels about four-fold at −40 mV (Figure 7E; Ctrl: 21.3 ± 3.2 pA/pF, SF0034: 68.7 ± 9.89 pA/pF) and about 25% at +40 mV (Figure 7F; Ctrl: 422.0 ± 52.1 pA/pF, SF0034: 542.4 ± 62.1 pA/pF). SF0034 treatment of cells co-expressing A337T subunits were also significantly increased at both −40 mV (Figure 7E; Ctrl: 4.7 ± 0.9 pA/pF, SF0034: 24.3 ± 5.7 pA/pF) and + 40 mV (Figure 7F; A337T Ctrl 200.6 ± 46.7 pA/pF, SF0034 266.7 ± 66.5 pA/pF). Current density in SF0034-treated A337T co-expressing cells was equivalent to that of untreated WT only expressing cells (n.s. between Kv7.2 + Kv7.3 controls versus A337T + Kv7.2 + Kv7.3 SF0034 at both −40 and +40 mV; results summarized in Supplementary Table S1).

## Discussion

Our analysis of human *KCNQ2* variants from severely affected individuals, with particular attention to effects of co-expression with WT subunits to mimic heterozygosity as is observed in people, serves two important purposes. First, finding a markedly abnormal functional profile supports a variant’s classification as pathogenic for a severe phenotype (Miceli et al., 2013; Orhan et al., 2014; Richards et al., 2015; Soldovieri et al., 2016). Second, characterization of novel variants giving severe phenotypes, even when heterozygous (i.e., expressed along with wild type subunits), builds an “intolerance map” of functionally critical sites within the protein structure and may highlight contributions to allosteric mechanisms that have been overlooked by hypothesis-driven experiments. Although heterozygous missense variants clustered near the CaM-binding Helix A and B of the intracellular CT domain account for approximately 21% of cases of *KCNQ2* encephalopathy (Millichap et al., 2016), studies to date have only begun to explain why very severe phenotypes result from some variants, while others cause benign familial neonatal epilepsy (Orhan et al., 2014; Soldovieri et al., 2016; Zhang et al., 2020). Understanding how these variants confer severe outcomes may aid development of targeted novel therapies.

Our analysis of Kv7.2 A337T and A337G, *de novo* missense variants within Helix A from patients with refractory seizures and developmental impairment (Saitsu et al., 2012; Millichap et al., 2016), yielded three main findings. First, these variants generated minimal or no detectable current when expressed alone and induced dominant-negative suppression when co-expressed with WT Kv7.2 (A337G) or with Kv7.2 and Kv7.3 (both A337T and A337G). The fractional reduction in conductance seen here was similar to that previously observed for T274M and G290D, two severe pore domain variants near the selectivity filter, and for R325G, a missense change at a proximal CT arginine residue that may contribute directly to PIP_2_ binding (Orhan et al., 2014; Soldovieri et al., 2016). Thus, our findings support the conclusion that these rare variants are pathogenic and provide further support for the idea that dominant-negative current suppression is an informative *in vitro* signature of severe EIEE phenotypes. Second, although A337G was not studied, trafficking of A337T to the AIS was normal in hippocampal neurons, as also shown recently for R325G and I205V (Soldovieri et al., 2016; Niday et al., 2017). In contrast, we showed that three pore domain mutations, a well-studied experimental mutation of the selectivity filter, G279S, and two of the most highly recurrent human *KCNQ2* encephalopathy variants (Millichap et al., 2016), T274M and A294V, strongly prevent AIS trafficking. These findings indicate that severe variants fall into at least two classes with respect to trafficking, a major difference in disease mechanism that has implications for therapy. It is unknown why mutations buried within the transmembrane ion pore would prevent AIS surface expression. It is intriguing to imagine that neuronal quality control mechanisms could distinguish between available and pore-blocked channels, and this deserves more study. Third, we showed that PIP_2_-dependent modulation was altered by both A337 variants, even though this residue, unlike the R325 studied by Soldovieri et al. 2016, lies within the CaM binding Helix A at a distance from any basic residue previously implicated in direct PIP_2_ interaction. Our PIP5K experiments suggest that treatments increasing PIP_2_ or making channels more responsive to PIP_2_ represent potential therapeutic approaches for these variants. Although the above listed conclusions are robust, our studies also exposed questions that are highly intriguing, interrelated, and for the present time, incompletely resolved, in spite of new related information on the structure of Kv7 channels obtained by cryoelectron microscopy (Sun and MacKinnon, 2020). These questions concern the impact of the variants on CaM binding, the kinetics of PIP_2_ association and dissociation from the channel, and on allosteric interaction between these two modulators.

### A337T CaM Binding Changes and Relevance to Pathogenicity

We found that protein half-life, total and surface protein abundance, and CaM binding were all reduced by the A337T mutation, but only when A337T subunits were expressed alone, i.e., without WT subunits. Protein stability effects were not seen for A337G, even though A337G strongly reduced current amplitudes. These rare variants, like nearly all of those found in *KCNQ2* encephalopathy, cause clinical symptoms in heterozygous individuals, so the observed effects on CaM binding and protein expression may not contribute to clinical severity. Alternatively, important effects on CaM binding may be insensitively detected in the transient expression systems used here and may be informatively brought to light by expressing the mutated subunit alone. Another example suggesting the potential predictive utility of data from variants expressed alone experiments is provided by a recent analysis of R144Q, R198Q, R201C, and R201H, *KCNQ2* voltage-sensor domain variants associated with severe encephalopathy phenotypes. These variants gave strong hyperpolarizing shifts in gating voltage-dependence if expressed alone but much smaller effects when co-expressed with WT subunits (Miceli et al., 2015; Millichap et al., 2016; Mulkey et al., 2017). Nonetheless, the markedly altered voltage-dependence exhibited by the variants when expressed alone correlated with the delayed age of onset of seizures and impaired development, distinctive aspects of the otherwise severe clinical phenotypes. Analysis of a larger number of pathogenic variants *in vitro*, and, potentially, studies of mouse models where the mutant alleles are driven by native promoters (Ihara et al., 2016), will clarify parameters for interpreting results obtained from variant-alone expression as part of efforts to assess pathogenicity and predict clinical severity.

### PIP_2_ Interaction Changes: Implications for Channel Allostery and Pathogenicity

PIP_2_ binding is obligatory for Kv7 channel activation. The conserved site mediating electromechanical coupling has been determined *via* cryoelectron microscopy of Kv7.1 in the PIP2-bound, open state (Sun and MacKinnon, 2020). Nonetheless, the dynamic interactions between PIP_2_ with the intact channel that result in macroscopic current properties remain very incompletely understood. These issues are potentially critical for learning how heterozygous variants can severely impair function within an assembled channel including both mutant and wild type subunits. Our experiments shed new light on PIP_2_ interactions with WT channels. WT Kv7.2 homomers gave about 4-fold smaller currents than WT Kv7.2 + Kv7.3 heteromers, but PIP5K co-expression enhanced homomer currents about 3-fold and heteromer currents only about 50%. Also, recovery from ciVSP-induced current inhibition followed a sigmoid time course well-fitted by a squared exponential function, as seen previously—an indication that binding of more than one PIP_2_ molecule is required for a channel to become active, as predicted by functional analysis (Falkenburger et al., 2010) and apparent in the new atomic structure. The ability of PIP5K co-expression to shift voltage-gating to the left, for both WT homomers and heteromers (Figure 6) suggests that elevated PIP_2_ concentration increased binding stoichiometry above levels minimally sufficient for voltage-gating. In sum, these results indicate that, under our recording conditions without PIP5K co-expression, PIP_2_ binding is not saturated and that Kv7.2 + Kv7.3 heteromers have higher affinity than Kv7.2 homomers, consistent with earlier single channel recordings (Hernandez et al., 2008).

Channels including A337T and A337G subunits gave reduced current amplitudes, whether the mutated subunits were expressed alone or co-expressed with WT subunits. The ability of M1 receptor or ciVSP activation to inhibit currents was reduced for heteromers containing mutant subunits, as if the mutations were occluding the PIP_2_ depleting effects of these treatments. In addition, when either A337T or A337G was expressed alone or with WT subunits, PIP5K co-expression strongly enhanced currents to levels equal to WT-only channels, and shifted gating to hyperpolarized potentials, as also seen for WT channels. These findings suggest that the channels including the A337 variants carry out electromechanical coupling similarly to WT channels, but bind less PIP_2_ under the basal conditions of our experiments. Further experiments, including recording of excised patches using application of PIP_2_ analogues to assess effects on single channel open probability and kinetics, could be used to test this interpretation.

The striking observations that A337T and A337G slow the kinetics of both current inhibition and current recovery during and after a ciVSP activation step (Figure 6; Supplementary Figures S8, S9) were unexpected. Those results could be explained if A337T and A337G exert their main effects not by altering PIP_2_ binding at the channel sites directly mediating electromechanical coupling, but instead, by delaying the migration of PIP_2_ between those sites and the bulk membrane. The Kv7.1 atomic structure shows conspicuous water-filled portals between the bulk cytoplasm and the intracellular mouth of the ion pore which includes patches lined by a number of basic residues (Sun and MacKinnon, 2017, 2020). It is noteworthy that additional basic residue sites between Helix A and B have been shown to functionally contribute to PIP_2_ interaction (Hernandez et al., 2008; Telezhkin et al., 2013; Kim et al., 2016a). These sites between Helix A and B are likely more peripherally located, but are not yet included in the known atomic structure. These basic sites may contribute to channel availability and modulation kinetics by maintaining an annular microdomain with an elevated local PIP_2_ concentration around the channel and helping to orchestrate migration bidirectionally.

Structures of the Helix A from Kv7.1, Kv7.3, and Kv7.4, co-crystallized with Helix B and bound CaM, show the conserved Ala residue (homologous to KCNQ2 A337) buried under and interacting with sidechains from the CaM C-lobe (Sachyani et al., 2014; Strulovich et al., 2016; Chang et al., 2018). In the intact channel, the four subunits’ Helix A-B/CaM modules form a ring below the transmembrane domain (Mruk et al., 2012; Sachyani et al., 2014; Alberdi et al., 2015; Sun and MacKinnon, 2017, 2020). CaM binding is highly dynamic (Mruk et al., 2012) and has been proposed to include multiple potential modes: binding to a Helix A alone, a Helix B alone, an A–B pair contributed by a single subunit, or crosslinked helices from two subunits (Alaimo et al., 2014; Sachyani et al., 2014; Alberdi et al., 2015; Chang et al., 2018; Sun and MacKinnon, 2020). Importantly, the Kv7.1 structure obtained with and without PIP_2_ bound reveals a large, nearly 180° flipping of the entire Helix A–B/CaM complex between the two states. How the large segment between Helix A and B participates in this state change is unknown. A337T and A337G are centrally located near the pivot point for this remarkable conformational flip. They may impair PIP_2_’s migration between the bulk membrane and the S4-linker binding site by altering the preferred CaM binding configuration, or by slowing the rate of the c-terminal conformational flip that follows PIP_2_ binding and unbinding. Evidence for dual impact on CaM and PIP_2_ was developed for another pathogenic variant, L637R, located much more distally in Helix D (Alberdi et al., 2015). There is already functional evidence that several modulatory signals, including elevated [Ca^2+^]i and phosphorylation by PKC and protein kinase CK2, may coordinately regulate the CaM configuration and PIP_2_ binding and thus, available current (Suh and Hille, 2008; Kosenko et al., 2012; Kosenko and Hoshi, 2013; Kang et al., 2014; Chang et al., 2018). It will be exciting to see a more integrated understanding of this refined and ancient mechanism emerge through future experiments.

Ezogabine, an activator of Kv7.2–5 subunit-containing channels that is approved for epilepsy in adults, has been shown to reverse effects of *KCNQ2* encephalopathy variants in patch clamp experiments (Miceli et al., 2013; Orhan et al., 2014; Soldovieri et al., 2016). Although ezogabine has also shown some benefits in seizure control in several *KCNQ2* encephalopathy infants, including an individual heterozygous for A337T (Weckhuysen et al., 2013; Millichap et al., 2016), side effects have limited its use. SF0034 is an ezogabine analog showing greater potency and selectivity in both cell-based and *in vivo* models (Kalappa et al., 2015). We found that SF0034 can increase macroscopic currents of A337T-containing channels, by some measures restoring current density levels to that of WT channels in the absence of drug. Novel Kv7 openers merit further study as a candidate therapy for *KCNQ2* encephalopathy due to loss-of-function variants, including those in the CT region that impair PIP_2_ modulation. Our A337T patient received ezogabine in the 6th month of life, with benefits for seizures but continued severe developmental impairment. Very early diagnosis and intervention may represent the best opportunity for favorable impact on development.

## Data Availability Statement

The raw data supporting the conclusions of this article will be made available by the authors, without undue reservation.

## Author Contributions

BT and Z-GJ performed all biochemistry, neuronal transfections, imaging, and electrophysiology experiments. BT and MX designed biochemical experiments. BT and ZGJ designed electrophysiology experiments and analyzed data. TT provided clinical information. BT and EC designed the project and wrote the manuscript. All authors reviewed and revised the manuscript, and approved the submitted version. EC funded the project.

### Conflict of Interest

BT, MX, ZGJ, and TT declare no competing financial interests. EC has served as a consultant to and as PI of investigator-initiated research grants from SciFluor Life Sciences, Knopp Biosciences, and Xenon Pharmaceuticals. This consultancy work and sponsored research was reviewed and approved according to the conflict of interest policies of Baylor College of Medicine.
