# Supplementary material for: Two KCNQ2 Encephalopathy Variants in the Calmodulin-Binding Helix A Exhibit Dominant-Negative Effects and Altered PIP2 Interaction
Source: Front Physiol. 2020 Sep 11;11:1144. doi: 10.3389/fphys.2020.571813 (PMC7518097; doi:10.3389/fphys.2020.571813)
Supplement: Supplementary file 1 [file Data_Sheet_1.pdf]

Supplementary Material

Two *KCNQ2* encephalopathy variants with dominant-negative effects exhibit altered PIP_2 and calmodulin interaction

Baouyen Tran[1], Mingxuan Xu[2], Zhi-Gang Ji[2], Tammy N. Tsuchida[3], and Edward, C. Cooper[1, 2, 4]

Departments of Neuroscience[1], Neurology[2], and Molecular and Human Genetics[4], Baylor College of Medicine, Houston, TX, 77030, United States
Departments of Pediatrics and Neurology[3], Children's National Medical Center, Washington, D.C. 20010, United States

Corresponding Author: Edward C. Cooper; Departments of Neurology, Neuroscience, and Molecular and Human Genetics, Baylor College of Medicine, One Baylor Plaza, M.S. NB302, Houston TX 77030; Tel: 713-798-4939; Fax: 713-798-3455; Email: ecc1@bcm.edu

Supplementary Figures

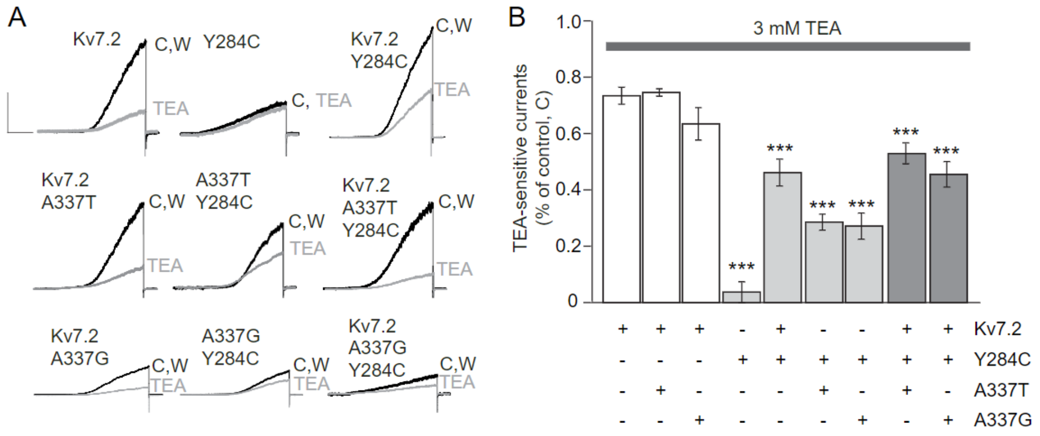

Supplementary Figure S1. A337 variant subunits can tetramerize with WT Kv7.2 and form channels. (**A**) Representative currents produced by the indicated subunit combinations before (C, control), during (TEA) and after washout (W) of 3 mM TEA (scales: 500 nA, 1 s). (**B**) Summary of results displaying percentage of TEA-sensitive currents compared to each control, where + indicates the presence of each subunit (***p<0.001 vs. Kv7.2 WT, Student's t-test, n = 9-12).

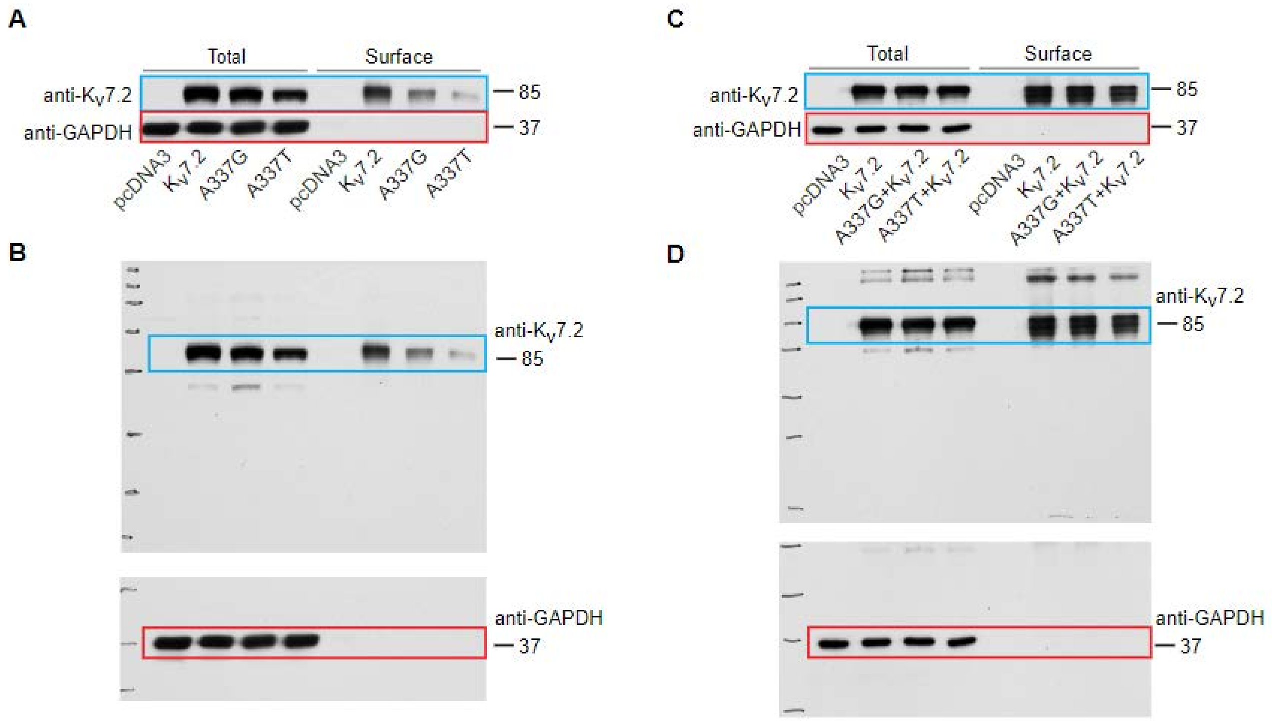

Supplementary Figure S2. Full-length blots of cropped images shown in Figure 1E, F. (**A**) Cropped western blot images of cell lysates and surface membrane biotinylated proteins from CHO cells transfected with WT Kv7.2, A337G, or A337T as shown in Figure 1E, taken from (**B**) full length blots probed for Kv7.2 and GAPDH. (**c**) Cropped western blot images of cell lysates and surface membrane biotinylated proteins from CHO cells co-transfected with WT Kv7.2 and A337G or A337T as shown in Figure 1F, taken from (**d**) full length blots probed for Kv7.2 and GAPDH. Blue boxes indicate Kv7.2 bands. Red boxes indicate GAPDH bands.

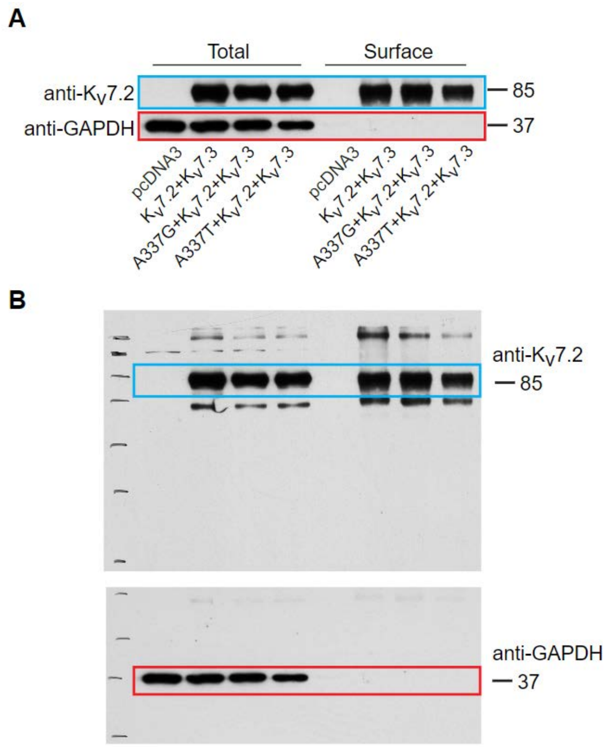

Supplementary Figure S3. Full-length blots of cropped images shown in Figure 2F. (**A**) Cropped western blot images of cell lysates and surface membrane biotinylated proteins from CHO cells co-transfected with WT Kv7.2+Kv7.3 and A337G, or A337T as shown in Figure 2F, taken from (**B**) full length blots probed for Kv7.2 and GAPDH. Blue boxes indicate Kv7.2 bands. Red boxes indicate GAPDH bands.

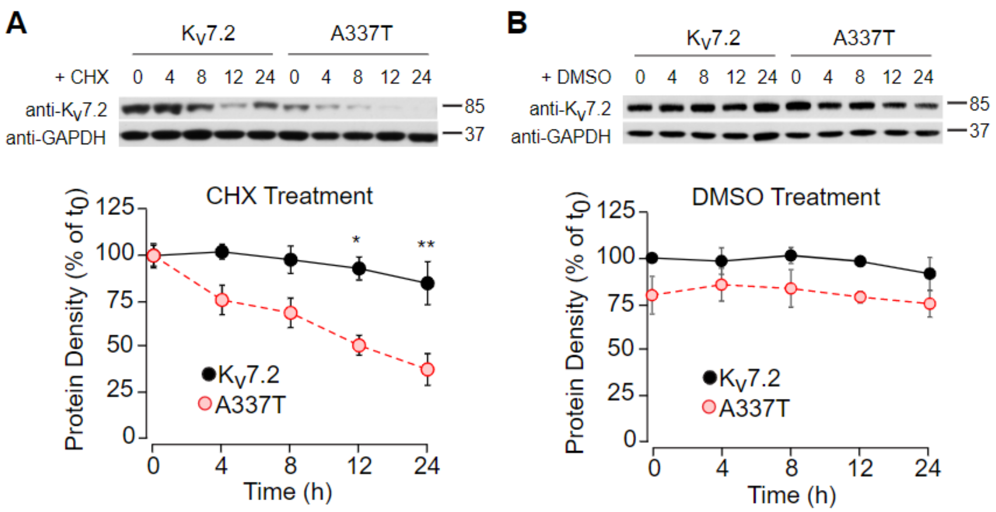

Supplementary Figure S4. A337T proteins expressed in HEK cells exhibited a reduced half-life. (**A, upper**) Western blot analysis HEK cells transfected with Kv7.2 or A337T. At 48 hours after transfection, the translation inhibitor, CHX (100 μg/ml, in 0.1% v/v DMSO vehicle) was added, and samples were taken subsequently for analysis. A337T proteins were more rapidly degraded, as displayed by progressive band density decrease over time. **(A, Lower)** Summary data: compared to the amount of each protein present at $t_{0,}$ A337T is significantly reduced at 12 h, (*p = 0.035) and at 24 h (**p = 0.003, One-way RM ANOVA, Tukey test, n = 6). (**B**, **upper**) Western blot analysis: control experiments using the vehicle (0.1% v/v DMSO) used for dissolving CHX in **A**. (B: Lower) Summary data: band density for each subunit, normalized to WT Kv7.2 at t_0. the level of protein expression did not change significantly over time compared to t_0, but there was a significant lower level of A337T in the starting material of versus WT Kv7.2 (**p = 0.006, One-way RM ANOVA, n = 6).

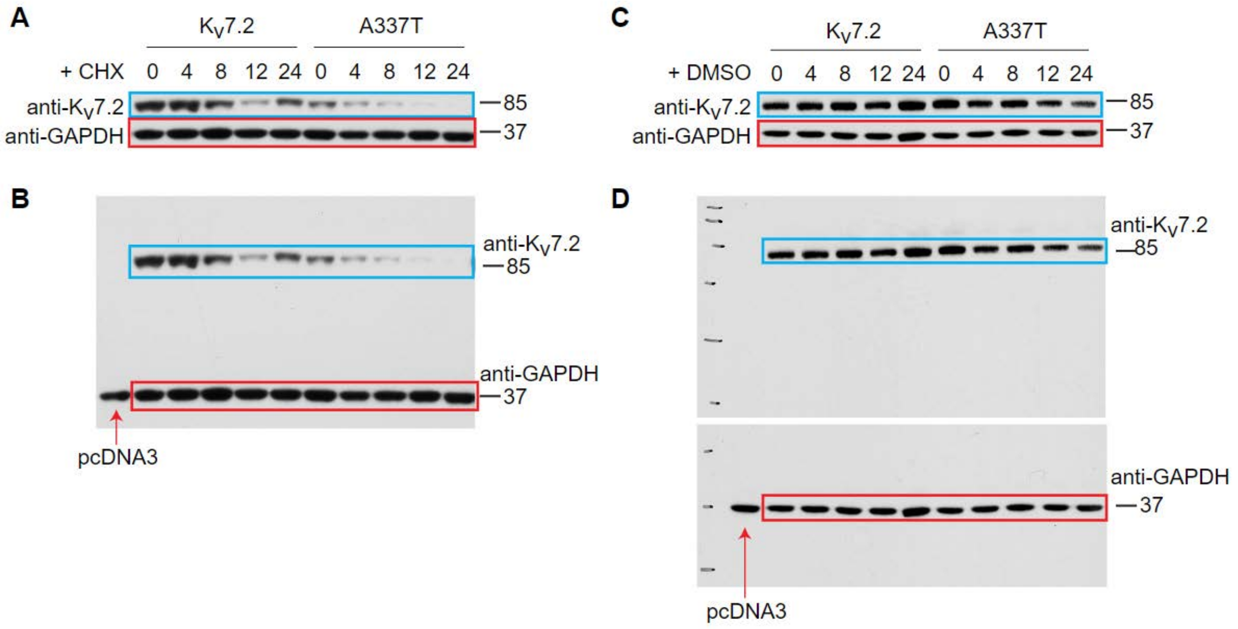

Supplementary Figure S5. Full-length blots of cropped images shown in Supp. Figure S4. (**A**) Cropped western blot images of cell lysates from HEK cells co-transfected with WT Kv7.2 or A337T and treated with CHX as shown in Figure S3A, taken from (**B**) full length blots probed for Kv7.2 and GAPDH. (**C**) Cropped western blot images of cell lysates from HEK cells co-transfected with WT Kv7.2 or A337T and treated with DMSO as shown in Figure S4A, taken from (**D**) full length blots probed for Kv7.2 and GAPDH. Blue boxes indicate Kv7.2 bands. Red boxes indicate GAPDH bands.

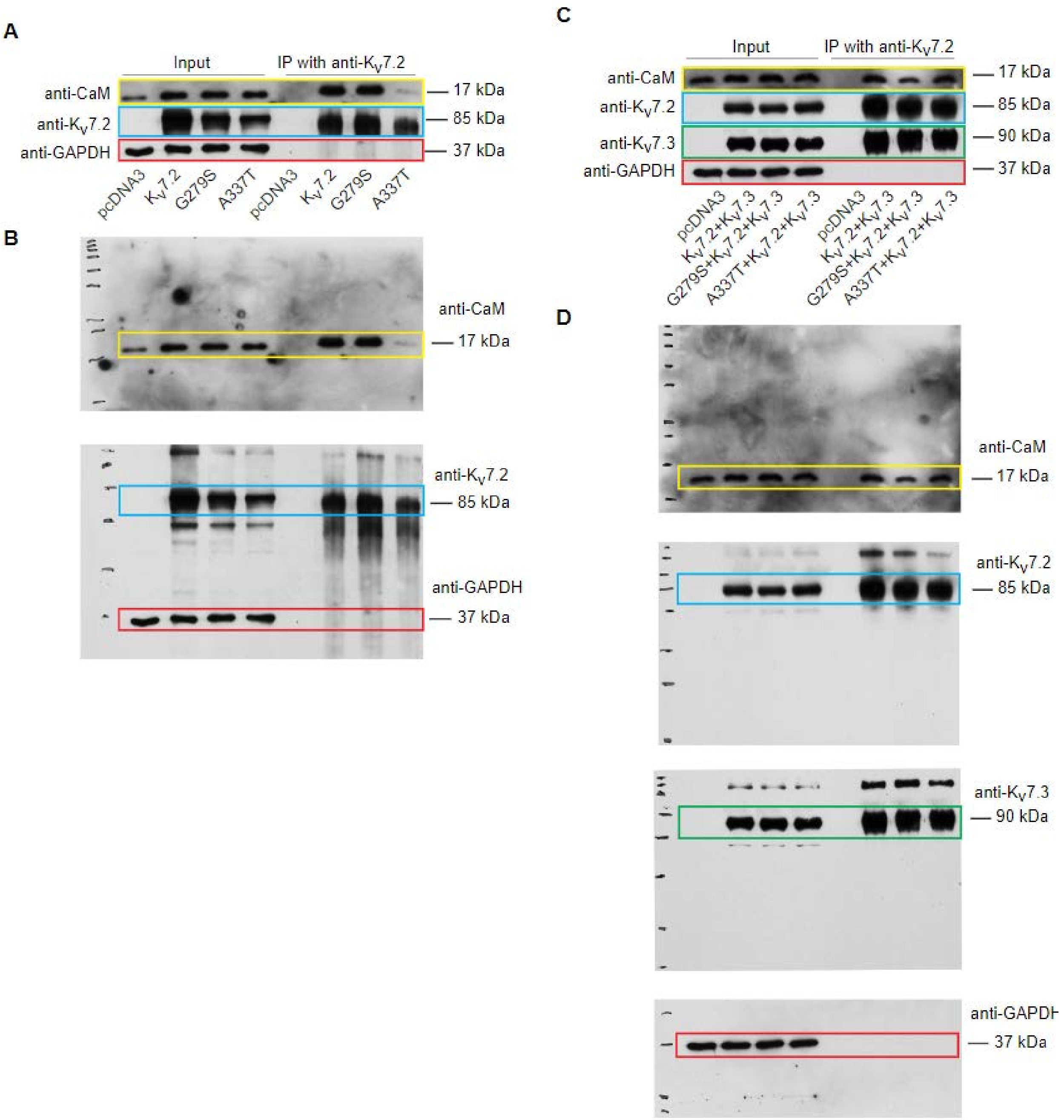

Supplementary Figure S6. Full-length blots of cropped images shown in Figure 3A, b. (**A**) Cropped western blot images of CaM levels after IP using anti-Kv7.2 antibodies from cell lysates expressing WT Kv7.2, G279S, or A337T as shown in Figure 3A, taken from (**B**) full length blots probed for CaM, Kv7.2, and GAPDH. (**C**) Cropped western blot images of CaM levels after IP using anti-Kv7.2 antibodies from cell lysates co-expressing WT Kv7.2+Kv7.3 and G279S or A337T as shown in Figure 3B, taken from (**D**) full length blots stripped and re-probed for CaM, Kv7.2, Kv7.3 and GAPDH. Blue boxes indicate Kv7.2 bands. Red boxes indicate GAPDH bands.

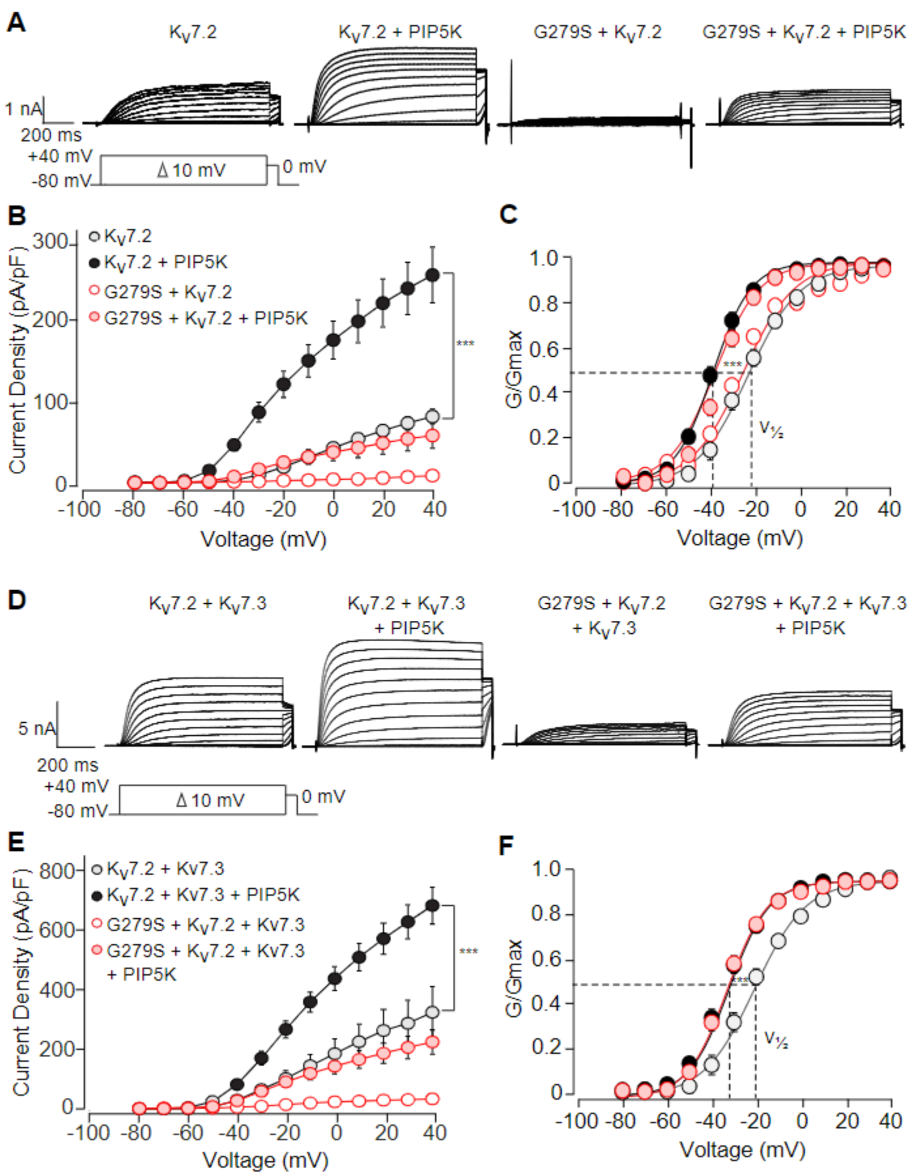

Supplementary Figure S7. PIP5K coexpression is unable to fully restore the current amplitude loss produced by G279S. (**A, D**) Representative currents produced by (**A**) WT Kv7.2 homomeric channels or (**D**) Kv7.2 + 7.3 heteromeric channels with or without the G279S variant expressed in ratios (1:1 and 1:1:2) to mimic heterozygosity. Where indicated, PIP5K was co-transfected (insets: voltage protocol). (**B, E**) Current-voltage plots show a significant increase of current density in wild type channels co-transfected with PIP5K in (**B**) homomeric and (**E**) heteromeric channels whereas with channels containing G279S, current density increases much less than seen for the WT or when A337T variants were present. (**C, F**) A left shift of the conductance-voltage curve is seen in both (**C**) homomeric and (**F**) heteromeric channels with co-transfection of PIP5K. (***$p<0.001$, Student's t-test, n = 5-12).

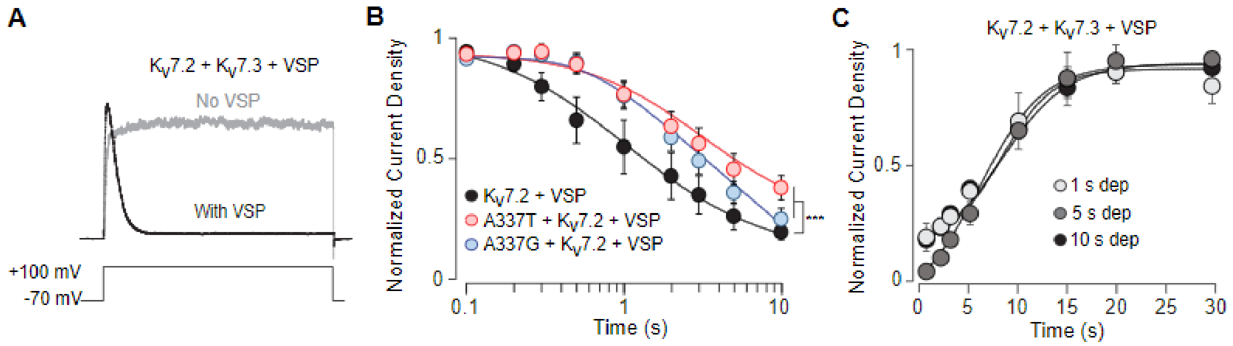

Supplementary Figure S8. Properties of Kv7.2 channels during PIP2 depletion and recovery. (**A**) Representative WT Kv7.2 + Kv7.3 currents, with or without co-transfection of ciVSP, evoked by step depolarization to +100 mV. Despite the long (10 s) strong depolarization, current levels are stable in the absence of VSP. (**B**) Normalized current densities from cells co-transfected with WT Kv7.2 only, or the indicated mutant and WT subunits (1:1 ratio), and ciVSP, using the pulse protocol shown in Figure 6A-C (***p <0.001, two-way ANOVA, Bonferroni test, n = 9-13).

(**C**) Quantification of recovery from ciVSP induced current inhibition for WT Kv7.2 + Kv7.3 after 1, 5, or 10 s depolarizations (n.s., two-way ANOVA, Bonferroni test, n = 4-13).

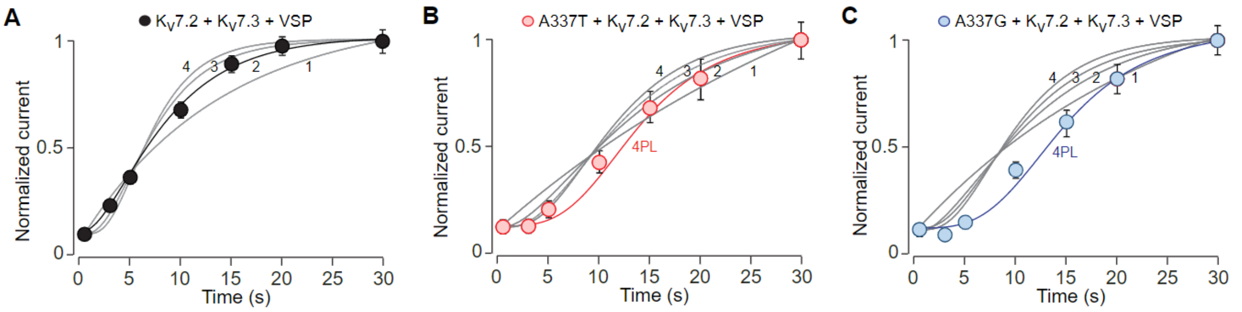

Supplementary Figure S9. Heteromeric channels containing A337 variants exhibit delayed onset of recovery from PIP_2 depletion. (A) Current recovery data from WT heteromeric channels was well-fit with a squared exponential function, as observed previously (Falkenburger et al., 2010). (**B, C**) In heteromers incorporating the A337 variants, recovery was more sigmoid, and was poorly fit by 1, 2, 3, or 4th power exponential equations. A 4 parameter logistic function provided a much closer fit of the for whereas channels containing variants.

Supplementary Tables

Supplementary Table S1. Current density and activation gating parameters of wild type or mutant heteromeric Kv7.2/7.3 channels after treatment with SF0034.

	**subunit**	**n**	**pA/pF (-40 mV)**	**pA/pF (+40 mV)**	**$V_{1/2}$, mV**	**k, fold mV/e**
Control	Kv7.2+Kv7.3	14	15.6±2.0	387.7±47.3	-23.8±0.6	10.7±0.7
	Kv7.2+Kv7.3+ A337T	14	4.5±0.9	171.2±43.0	-34.5±0.6	10.5±0.7
SF0034	Kv7.2+Kv7.3	14	67.9±10.6	525.2±63.6	-23.0±0.4	8.9±0.4
	Kv7.2+Kv7.3+ A337T	14	27.2±6.8	255.6±62.2	-32.5±0.4	9.6±0.5

Supplementary Reference

Falkenburger, B.H., Jensen, J.B., and Hille, B. (2010). Kinetics of PIP2 metabolism and KCNQ2/3 channel regulation studied with a voltage-sensitive phosphatase in living cells. *J Gen Physiol* 135**,** 99-114.
